# Compositional maturation of the microbiome and adaptive immunity in the postnatal period

**DOI:** 10.3389/fimmu.2026.1772425

**Published:** 2026-05-05

**Authors:** Miranda Green, Shane Cleary, Bryce Kwiecien-Delaney, Jane A. Foster

**Affiliations:** 1 Department of Psychiatry and Behavioral Neurosciences, McMaster University, Hamilton, ON, Canada; 2 Center for Depression Research and Clinical Care, Department of Psychiatry, O'Donnell Brain Institute, University of Texas Southwestern Medical Center, Dallas, TX, United States

**Keywords:** compositional data analysis, gut-brain axis, lymphocytes, microbiome, T-cell, postnatal development, microbiome-immune

## Abstract

**Introduction:**

Recent research has highlighted the role of the gut microbiome in shaping the development and function of the mammalian immune system. Interactions between these complex networks of microbes and host cells serve not only to train major aspects of adaptive and innate immunity but also to establish commensal host-microbe relationships and symbiosis throughout the lifespan. T-cells are a critical aspect of this paradigm, acting as intermediates between the microbiome and many aspects of host health and disease. Despite a large body of literature examining these interactions, we have yet to completely understand how the ontogeny of these systems co-evolves across the lifespan and how the emergence of specific T-cell-microbe signals relates to key developmental milestones.

**Methods:**

To answer this question, this work conducted a compositional integrative analysis on deep immune and microbiome profiling of wild-type C57Bl/6 mice across the first two weeks of life, post-weaning, and young adulthood.

**Results:**

The results show that T-cell ontogeny follows different developmental trends in mucosal and peripheral immune compartments and that temporal trends in microbial community abundance creates a modular network of associations between specific taxa and functional T-cell subsets.

**Discussion:**

These results provide insight into the longitudinal development of microbiota-immune system interactions throughout the lifespan, as well as the mechanistic relevance of microbiota-derived signals at key developmental milestones.

## 1 Introduction

The role of the microbiome in host development in the context of many physiological systems and pathologies, ranging from gastrointestinal health to psychiatric disorders has emerged as an important research topic (1, 2). The bidirectional exchange of immunomodulatory signals between microbiota and host at the intestinal barrier promotes immune cell development, influences immune function, fortifies anti-pathogen responses, and simultaneously enables colonization and maturation of commensal microorganisms that contribute to host health (3–7). Evidence from preclinical and clinical studies have highlighted how interactions between the microbiome and the host serve to train and tune the developing immune system (8–12). Postnatal colonization of the gut with commensal microbes allows for immune education and tolerance in a rapidly changing environment (11, 13, 14). Microbiota-immune signaling is a key gut-brain axis pathway by which the microbiome influences brain development and function. Notably, microbiome colonization and adaptive immune development overlap with a formative window of brain wiring and circuitry formation, creating an opportunity for microbiota-immune-brain signaling to directly influence neurodevelopmental outcomes (15–17).

An increasing body of evidence demonstrates that cross-talk between microbes and T cells influence brain development and behavior in mouse models (18–20). T-cell deficient (*TCRβ-/-δ-/-*) mice, which lack functional T-cells due to a double knockout of critical T-cell receptor subunits, display reduced anxiety-like behavior (20), similar to behavioral changes observed in germ-free (GF) mice, lacking all commensal microbes (21–24). Behavioral differences due to immune deficiency emerge very early in life, as *TCRβ-/-δ-/-* pups show developmental delays in the righting reflex in the first week of life compared to wildtype C57Bl6 (B6) controls (18). Recent findings demonstrate that T-cell deficient mice harbor an altered microbiome composition with altered functional output compared to B6 mice (19). Through cross-sectional multi-omic phenotyping, the same study demonstrated that T-cell deficiency impacts the developmental trajectory of the gut microbiome along with the gut metabolome and brain neurochemistry over postnatal development (postnatal day [P] 17 to young adult), resulting in a unique longitudinal signature of host immune status (19). Evidence of immune deficiency impacting the microbiome has also been observed in severe combined immunodeficient (SCID) and non-obese diabetic SCID (NOD/SCID) mice, lacking mature B- and T-lymphocytes, as well as T-cell deficient CD3-epsilon–/– mice (25, 26). Thus, understanding the role of T-cells in coordinating communication between the microbiome and other host systems is of particular importance, especially in the context of coordinating of microbe-driven developmental processes in early life.

The present study aimed to map how the microbiota and host immune cell repertoires co-develop during early postnatal period in B6 mice. To achieve this, the present experiment performed immunophenotyping of lymphocyte populations in the gut, including lamina propria and intestinal epithelium, and immune organs, including thymus and spleen, during postnatal development in parallel with profiling of gut microbiota within the same animals. Integrating this multi-modal data using a compositionally robust analysis pipeline revealed a unique developmental signature of microbe-immune interactions across early life.

## 2 Materials and methods

### 2.1 Animals and tissue isolation

C57Bl/6 (B6) mice were initially purchased from Charles River (Kingston, USA) and bred in-house at St. Joseph’s Healthcare animal facility. The mice were maintained in specific-pathogen-free (SPF) housing in sanitized cages with filter bonnets under a 12 h light–12 h dark cycle, with lights on at 5 AM. Food and water were available ad libitum. At weaning (post-natal day [P] 21), pups were caged by sex with 2–4 littermates per cage. All mice were drug- and test-naïve and weighed prior to dissection. All experimental procedures were approved by the Animal Research Ethics Board of McMaster University, in accordance with the guidelines of the Canadian Council on Animal Research. Biological replicates (n = 6) were collected per sex per timepoint (pre-weaning: postnatal day [P]7 and P14; post-weaning: P24 and 8–9 weeks [8W]). Fecal, cecal, IEL and LPL replicates for pre-weaning timepoints (P7 and P14) comprised pooled samples from 2–3 sex-matched littermates (n = 54 mice total) but were treated as a single sample for statistical purposes.

Adult mice and pups were killed by decapitation, and the entire small intestine, from the pyloric sphincter to the ileocecal junction, was removed from the abdomen. The small intestine (SI) was used in order to collect enough immune cells for analyses. Notably, there are significantly more intraepithelial lymphocytes in the SI compared to the colon. The lamina propria was also isolated from the small intestine. After removal of Peyer’s patches and lymphoid aggregates, the intestinal contents and mucus were removed by flushing with cold phosphate buffered saline (PBS) and gentle mechanical propulsion with tweezers. The intestines from P7 and P14 mice were cut into 5-mm pieces, whereas those from P24 and 8W were opened lengthwise and gently scrubbed on a paper towel moistened with harvest media to remove excess mucus. Processed intestine was stored in cold harvest media until lymphocyte isolation began. In parallel, spleen and thymus tissue were collected and stored in cold, sterile PBS. Colon and cecal contents were collected and frozen on dry ice.

### 2.2 Spleen and thymus lymphocyte isolation

Spleen was transferred to cold PBS in a 35 mm dish for additional fat trimming and visual inspection. Using the flat end of the plunger from a 5mL syringe, the spleen tissue was then crushed to release splenocytes. After homogenizing splenocytes in the PBS with a serological pipette, they were filtered through a primed 100 μm cell strainer into a sterile 50mL falcon tube. The strainer was then washed with cold PBS and discarded. The tube containing cell suspension with was then topped up with PBS, until ~1/2 full, then centrifuged at 300 x g for 10 minutes. After removing the supernatant, the pellet was resuspended with 5mL 1X Red Blood Cell Lysis Buffer and Incubated on ice for 4–5 minutes with occasional shaking. After incubating, the reaction was stopped by diluting the Lysis Buffer with 20–30 ml of 1X PBS. Cell suspension was once again centrifuged (350 x g) and the supernatant discarded, and one additional wash was performed before resuspending the pellet in FACS buffer to a final concentration of approximately 1M cells/mL.

To release thymocytes from tissue, the thymus was crushed through a 70 μm primed cell strainer on a sterile 50 mL falcon tube using the plunger of a sterile 3mL syringe. Strainer was then rinsed with 3mL PBS. To increase yield, remaining thymus pieces were smashed through the mesh a second time followed by another PBS rinse. Finally, the sample was Centrifuged at 400 × g for 4 min at 4 °C, the supernatant was discarded and thymocytes were resuspended with FACS buffer to a final concentration of approximately 1M cells/mL. All cell suspensions were kept on ice prior to staining.

### 2.3 Isolation of intestinal lymphocytes

Intestinal intraepithelial lymphocyte (IEL) suspensions were obtained from one to three animals depending on their postnatal age. In P7 and P14 mice, two or three intestines of mice from the same litter were combined into a single tube and processed simultaneously, whereas intestinal tissues were processed individually for P24 and 8W mice. IELs and lamina propria lymphocytes (LPLs) were isolated and processed as previously described (27), with some modifications to the protocol. In brief, intestinal pieces were first washed three times in harvest media, then transferred into a DTT solution (1 mM dithiothreitol [DTT], 10 mM 4-(2-hydroxyethyl)-1-piperazineethanesulfonic acid [HEPES]) and incubated in a shaker at 37 °C at 220 rpm for 20 min. This process was repeated twice. Filtering, pooling and spinning down supernatant after each incubation was performed to isolate a combined IEL population. Remaining tissue was then incubated with ethylenediaminetetraacetic acid disodium salt dihydrate (EDTA) solution (30 mM EDTA, 10 mM HEPES) for two 30-minute periods at 37C, followed by a final wash with fresh harvest media. Tissue was next digested using a 10 mM collagenase solution at 37 °C for 40–45 mins to extract LPLs. After resuspension, both IELs and LPLs were purified using a 44/67% density gradient (RPMI 1640 medium & Percoll) and resuspended in FACS wash (10% bovine serum albumin [BSA], 2mM EDTA) for counting and viability assessment using trypan blue exclusion dye.

### 2.4 Cell staining and flow cytometry

After counting, cells were aliquoted at volumes of 100 uL and concentrations ranging from 0.5–2 M cells/mL depending on yield and age of the mouse, with a minimum of two aliquots per sample. Aliquots from a given sample were combined with 50 uL of the desired surface stain marker cocktail (**Supplementary Table 1**) and incubated at room temperature for 30 min. After subsequent centrifugation and washing of pellets, cells were resuspended in 1X Foxp3 fixation/permeabilization solution (eBioscience) for 15–16 h at 4C. Following overnight fixation cells were spun down and washed twice using 1X permeabilization buffer (eBioscience). Pellets were subsequently resuspended in 50 μL of intracellular staining cocktail (**Supplementary Table 1**) for 50–60 min at 4C. After another 2 washes, cells were then resuspended in 250 μL FACS wash and filtered at 70 μm to remove aggregates. Flow cytometry was conducted using the BD FACSCelesta instrument (BD Biosciences). Between 50,000 and 1M gated events were acquired for each sample, based on the forward/side scatter properties of the cell preparations and negative viability stain (live cells). Data was collected using the FACSDiva software and analyzed in FloJo version 10.7.

### 2.5 Flow cytometry gating and analysis

In all panels, the gating process first involved lymphocyte identification based on FSC-A and SSC-A characteristics, followed by doublet exclusion based on FSC-A/FSC-H as well as SSC-A/SSC-H characteristics. Cell viability was assessed after gating on CD45+ cells as a pan-lymphocyte marker, with a minimum cell viability of 85% in experimental samples. Depending on the panel, further gating was performed to assess functional T-cell subsets within CD4+CD8- or CD3+CD19- populations (details shown in **Supplementary Figures S1**–**S4**). All functional cell populations (FoxP3(+/-) and RORγT(+/-)) were gated based on internal, age-matched fluorescence minus one (FMO) control. Flow cytometry data were initially analyzed using the FlowJo software and then exported for further analysis and multi-omic integration in R. Data was normalized to report each population as a percentage of (%) CD45+ cells, % T-cells or % Parent depending on the marker(s) in question, while dot plots and additional visualizations were used to check for consistency between samples at each time point.

### 2.6 Microbiome sequencing and curation

Prior to DNA extraction, sex-matched fecal and cecal samples from P7 and P14 littermates (ID pairs matching pooled intestines) were pooled together to increase combined mass over 15mg. Microbiome DNA extraction and sequencing were performed by Microbiome Insights (British Columbia, Canada). DNA was extracted using the Qiagen MagAttract PowerSoil DNA KF kit (Formerly MOBio PowerSoil DNA Kit) using a KingFisher robot. DNA quality was evaluated visually via gel electrophoresis and quantified using a Qubit 3.0 fluorometer (Thermo-Fischer, Waltham, MA, USA). Libraries were prepared using an Illumina Nextera library preparation kit with an in-house protocol (Illumina, San Diego, CA, USA). Paired-end sequencing (150 bp x 2) was done on a NovaSeq. Shotgun metagenomic sequence reads were processed with the Sunbeam pipeline. Initial quality evaluation was done using FastQC v0.11.5 (28). Processing took place in four steps: adapter removal, read trimming, low-complexity-reads removal, and host-sequence removals. First, adapter removal was done using cutadapt v2.6 and trimming was performed with Trimmomatic v0.36 (29) using custom parameters (LEADING:3 TRAILING:3 SLIDINGWINDOW:4:15 MINLEN:36). Low-complexity sequences were detected with Komplexity v0.3.6 (30). High-quality reads were mapped to the human genome (Genome Reference Consortium [GRC] Human Reference 37) to account for human manipulation of the samples as well as to the mouse reference genome (GRCm38.p6) for detection of host reads; reads that mapped to either reference were removed from the analysis. The remaining reads were taxonomically classified using Kraken2 with the PlusPF database from 2021-01-17 (31). For functional profiling, high-quality (filtered) reads were aligned against the SEED database via translated homology search and annotated to Subsystems, or functional levels, 1–3 using Super-Focus (32).

### 2.7 Statistical analysis

#### 2.7.1 Microbiome data processing

Preprocessed microbiome data was imported into R version 4.1.0 and assembled into a phyloseq object for downstream analysis. Subsequent diversity analysis was conducted using the raw (unfiltered, unmerged) microbiome data. Alpha diversity metrics included the Inverse Simpson and Shannon index as well as observed reads (richness); linear models for each metric were constructed using the lm() function in R to model observed trends in alpha diversity as a function of timepoint and sex. Beta diversity between samples was explored using principal coordinate analysis (PCoA) with Jaccard, Bray–Curtis, (Manhattan) and Aitchison distance metrics applied to microbiome relative abundance data. To assess the influence of timepoint group on microbiome composition, permutational multivariate analysis of variance (PERMANOVA) was conducted using the function *adonis2()* from the phyloseq package (33), and pairwise comparisons were implemented to assess drivers of omnibus group differences significant to p < 0.01. To validate and account for bias in initial alpha and beta diversity measures, the breakaway package was implemented to estimate unobserved taxonomic richness across samples and repeated the above-described statistical tests for comparison (34).

Prior to differential abundance (DA) analysis, microbial sequence tables were further preprocessed using the retain resolve method (https://rdrr.io/github/SarahAsbury/retainresolve), as previously described (18, 35). In brief, taxa were sequentially filtered and glommed to obtain a final dataset that retains biologically significant taxonomic classifications while removing noise in the form of spurious sequences and highly partitioned subgroups. cDA analysis was executed using a consensus technique, combining models constructed using Linear models for Differential Abundance analysis (LinDA) (36) and Analysis of Compositions of Microbiomes with Bias Correction 2 (ANCOMBC-2) with the same fixed effects formula. All taxa were assessed for significant linear and non-linear abundance trends across time points using weighted polynomial contrasts, followed by pairwise comparisons to determine the contribution of consecutive developmental periods to globally significant results. To account for compositionality, data were either centered log-ratio or log-ratio transformed prior to mean comparisons. Criteria for significantly different taxa between groups were set at a Benjamini–Hochberg (BH) corrected p-value < 0.01 and an effect size analog (log-2-fold change or change coefficient) of >1. A final list of consensus taxa was then constructed using the mutually significant taxa between models.

#### 2.7.2 Flow cytometry analysis

Flow cytometry data is inherently compositional since each observation can be transformed into a vector of proportions of each type of immune cell being quantified and the total proportions for each type of T-cell within a given level of the hierarchical gating structure should sum to 1. Historically, such properties have often been ignored: populations of interest were directly compared with percentages treated as continuous variables and analyzed using standard statistical tools such as t-tests and ANOVAs. This is problematic as it ignores a number of additional properties introduced by a hierarchical nested data structure, including perfect or strong negative correlations between complementary subsets and other structural associations between variables imposed by the sum constraint (37, 38). In light of these unique data features, two approaches were combined: nested and un-nested Dirichlet regression. Dirichlet regression is an extension of a general linear model (GLM) framework adapted for compositional data. It is a multivariate extension of the beta distribution, which can accurately and flexibly model the unique distribution of data constrained within the range [0,1] (39). The nested Dirichlet model further extends multi-group analysis from a single compositional space into a tree-based framework, where the terminal nodes sum to 1 (39, 40). These values can also be expressed as proportions of a subtree rooted at the parent node, whereby sub-trees on different branches of the global tree are conditionally independent of the others. Importantly, the nested Dirichlet distribution relaxes the assumptions of its unnested counterpart, such that mean-variance scaling and negative covariance between variables is not required. This allows for independent testing of sub-trees that can be used to derive a global test statistic for the tree spanning all variables. Fortunately, in the case of flow cytometry data, the nesting tree is objectively derived from the gating strategy, removing the need for a data-driven tree-finding algorithm. However, when more than 2 populations are present (e.g., a quadrant gate) this does not guarantee a negative correlation between all variables at each level of the nesting tree. Thus, testing for changes in lymphocyte subpopulations over time implemented either beta regression (2 populations) or Dirichlet regression (>2 populations) using the proportions of each lymphocyte within the parent gate as a response variable. For non-binary models (i.e., 3 or more populations), correlations between all response variables were calculated to ensure interdependency assumptions were met. Where positive correlations were observed between a subset of components, further partitioning of the data tree was performed to account for nesting structure and tests were conducted on each layer of the nesting tree (40). Similarly, for the global tests, a common mean vector π is assumed for all groups under the null hypothesis, and an LRT statistic Λ_overall_ is calculated by summing test statistics from individual regressions performed for each sub-tree (i.e., a given level of the gating tree) of the data. Regressions were conducted using either the DirichReg (41) or betareg (42) packages in R, the former using an *alternative* parameterization to model marginal Dirichlet distributions for populations of interest with separate mean and precision estimates (additional details described in the package vignette and Maier (41)). Global tests for the effect of Timepoint were conducted using weighted polynomial contrasts; following a significant global test, individual components were regressed against Timepoint using reverse difference contrasts (e.g. comparing sequential timepoints) to inspect their contribution to overall compositional differences over time. As with microbiome data, all results were adjusted for multiple comparisons using the BH method within each tissue, with an FDR < 0.05 considered significant.

#### 2.7.3 Integrated microbiome-immune analysis

To conduct an integrated analysis combining flow cytometry and microbiome data, each dataset was first transformed to account for their unique compositional properties that would make them amenable to downstream analysis. Although transformed data can offer limited interpretability compared to a specialized regression framework such as the Dirichlet regression described above, transformation and stabilization were preferable in this context to examine inter-dataset relationships and their relation to developmental processes. Retain-resolved, CLR-transformed microbiome data was used for this analysis, along with flow cytometry data that was normalized using the three-step unzeroing, stabilization and standardization process previously described (37). The transformed, standardized data were scanned for artifacts, influence points, and potential outliers before proceeding. Immune cell features used in this analysis are listed in **Supplementary Table 2**. Covariation between multi-omic datasets were quantified using a Mantel test, which calculates the Pearson correlation between distances of two matrices. In this case, we used the Aitchison distance matrix for microbiome data and Euclidean distance applied to normalized flow cytometry data. Inter-sample dissimilarity matrices were then compared using the *mantel()* function in the vegan package, stratified by sex as a covariate (partial Mantel test); P-values were derived based on 1000 permutations and an FDR < 0.05 was considered statistically significant.

Next, to further explore specific immune cell-microbe associations, an omnibus network analysis was performed on CLR-transformed microbiome and flow cytometry data using the propr R package (43). Proportionality offers a valid, compositional data analysis (CoDA)-based alternative to correlation, which relies on log-ratio variance (LRV) to measure the coordination in expression/abundance of a given pair of compositional parts (44, 45). Not only has this method been shown to be robust to spurious correlation (44) but also outperforms standard tools that are not scale-invariant when applied to multi-omic data (46). Although the range of the proportionality measure ρ ranges between -1 and 1, the interpretation of negative values is limited given reciprocal events are not robust with respect to the choice of reference (43). To account for this, we also computed pairwise Spearman correlations on the separately CLR-transformed microbiome and immune cell datasets, which allows for negative relationships between network features. To reduce the overall testing burden, our analysis was restricted to developmentally regulated consensus taxa that were significantly differentially abundant between adjacent time points in our cDA analysis (P7 vs P14, P14 vs P24, P24 vs 8W; full list of taxa provided in **Supplementary Table S3**), as well as removing extraneous double-negative and redundant populations from the transformed immune cell data (ex. RORγT-CD25+ and FoxP3-CD25+ cells measured under the same parent). For proportionality, the *updateCutoffs()* function was used to filter out significant values with a proportionality coefficient (ρ) > 0.45, corresponding to an FDR < 0.02 based on 1000 network permutations. For Spearman correlations, pairwise relationships at r > 0.6 and FDR < 0.01 were used for network construction. Pairwise scatter plots were generated for all significant results to remove false positives or relationships driven by single outlier values. The resulting adjacency matrix was extracted and used to construct an undirected graph using the *graph_from_adjacency_matrix()* function before further processing and visualization using the igraph package (47) and Gephi version 0.10 (48).

Next, we implemented a random-matrix theory (RMT)-based approach to validate the complex network structure between datasets (49). Specifically, 1000 random networks with the same node and edge count as each empirical matrix were generated using the Erdös-Réyni algorithm, and average topological features of these random networks, including average path length (APL), average clustering coefficient (ACC), and modularity, were calculated. Averaged results for these indices across all random networks were then compared to values obtained from the corresponding observed network using a statistical Z-test.

Finally, correlation/proportionality matrices (r) were converted to dissimilarity matrices (d) using the formula:


$$d=\;\sqrt{2(1-r)}$$

As implemented in the cor2dist() function in the psych R package (50). Hierarchical clustering was performed to assess cluster stability and network modularity using Ward linkage via the *hclust()* function from the dendextend package (51). Inter and intra-cluster connectivity for each network type was then assessed using the *generate_cluster_cor_mat()* function from the NetCluster package (52). Cluster number was determined based on a minimum correlation of by-cluster matrix with the observed correlation matrix of 0.85.

## 3 Results

### 3.1 Distinct developmental trajectories were observed for gut mucosal and systemic T-cell populations in the postnatal period

**Figure 1:**
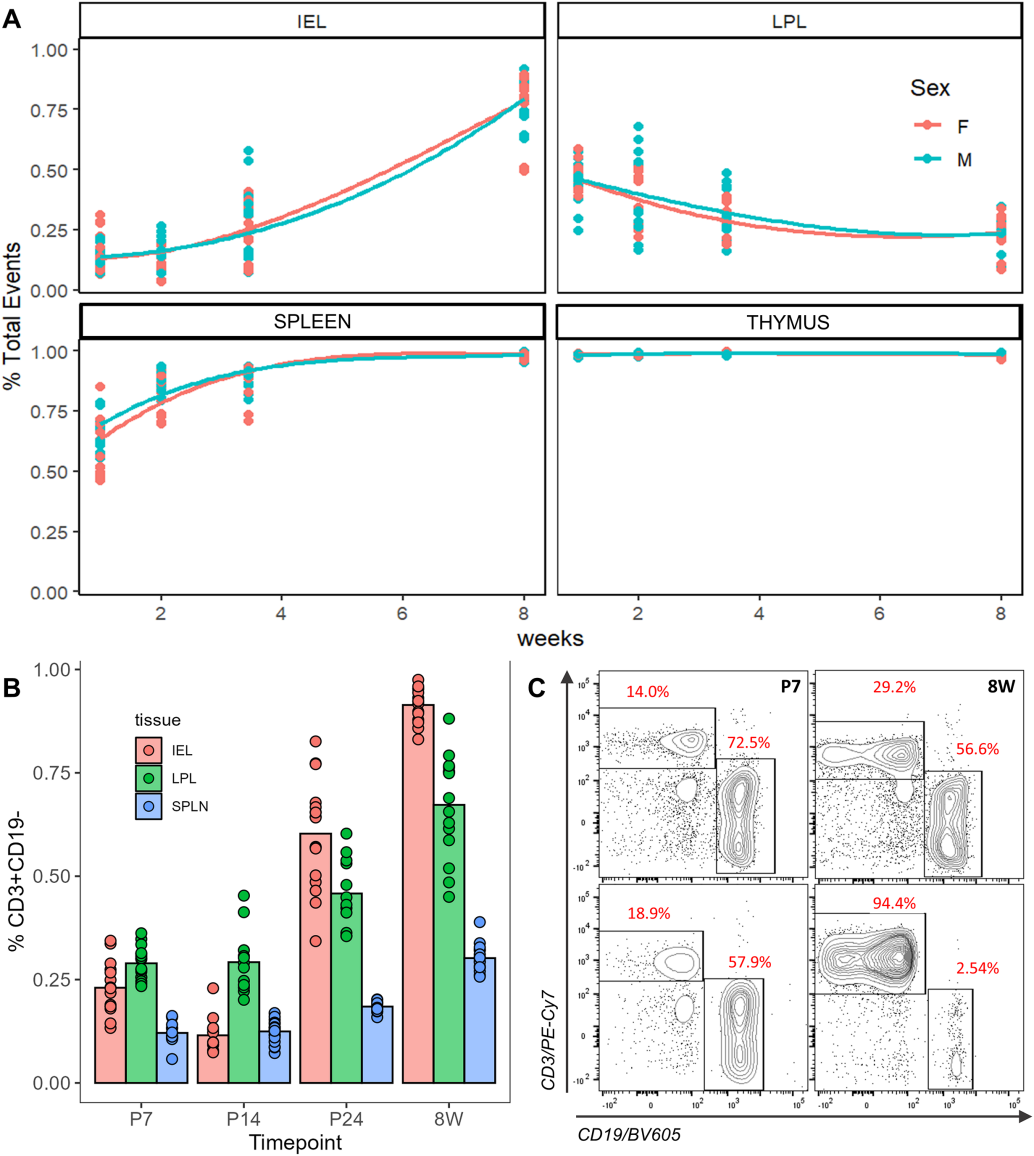
Overview of total lymphocytes across the postnatal period. **(A)** Percentage of CD45+ cells as a fraction of total single-cell events in mucosal (top row) and primary immune tissue (bottom row) colored by sex. **(B)** Total fraction of CD3+CD19- T-cells as a fraction of live CD45+ events in relevant tissues for each timepoint (n = 12 per group). **(C)** Cell gating on exemplary samples showing expansion of CD3+ T-cell prevalence in spleen (top row) and IEL (bottom row) compartments from P7 (left) to 8W (right).

To map expansion and diversification of the T-cell and immune cell repertoire across the postnatal period, the abundance of T-cell populations from P7, P14, P24 and 8W male and female B6 mice were quantitated in the thymus, spleen, and intestine (intraepithelial) lymphocytes (IELs), and laminar propria lymphocytes (LPLs). Tissue patterns for the total proportion of CD45+ lymphocytes over time are shown in **Figure 1A**. In the thymus, the total CD45+ populations remained relatively constant over the postnatal and postweaning period, while in the spleen there was an increase during the second week of life that persisted into adulthood. In the LP, there was a reduction over postnatal development. In the IEL samples, it was not until post-weaning that relative populations of CD45+ cells increased, observed here at P24 and 8W, indicating a distinct trajectory of proliferation in each of the immune compartments. As illustrated in **Figure 1B**, tissue compartments also showed unique developmental differences in the proportion of CD3+ T-cells across the measured timepoints. While thymocytes were consistently comprised of 97-99% CD3+ cells (data not shown), all other tissues showed an increase in the proportion of T-cells over time, with the most notable increases in population abundance between P14 and P24. By 8W, spleen tissue showed the lowest relative T-cell population (30%) and the smallest population increase from early life to adulthood, while IEL T-cell populations expanded from a mere 21% at P7 to nearly encompassing the entire lymphocyte pool (93%) at 8W (**Figure 1C**).

**Figure 2:**
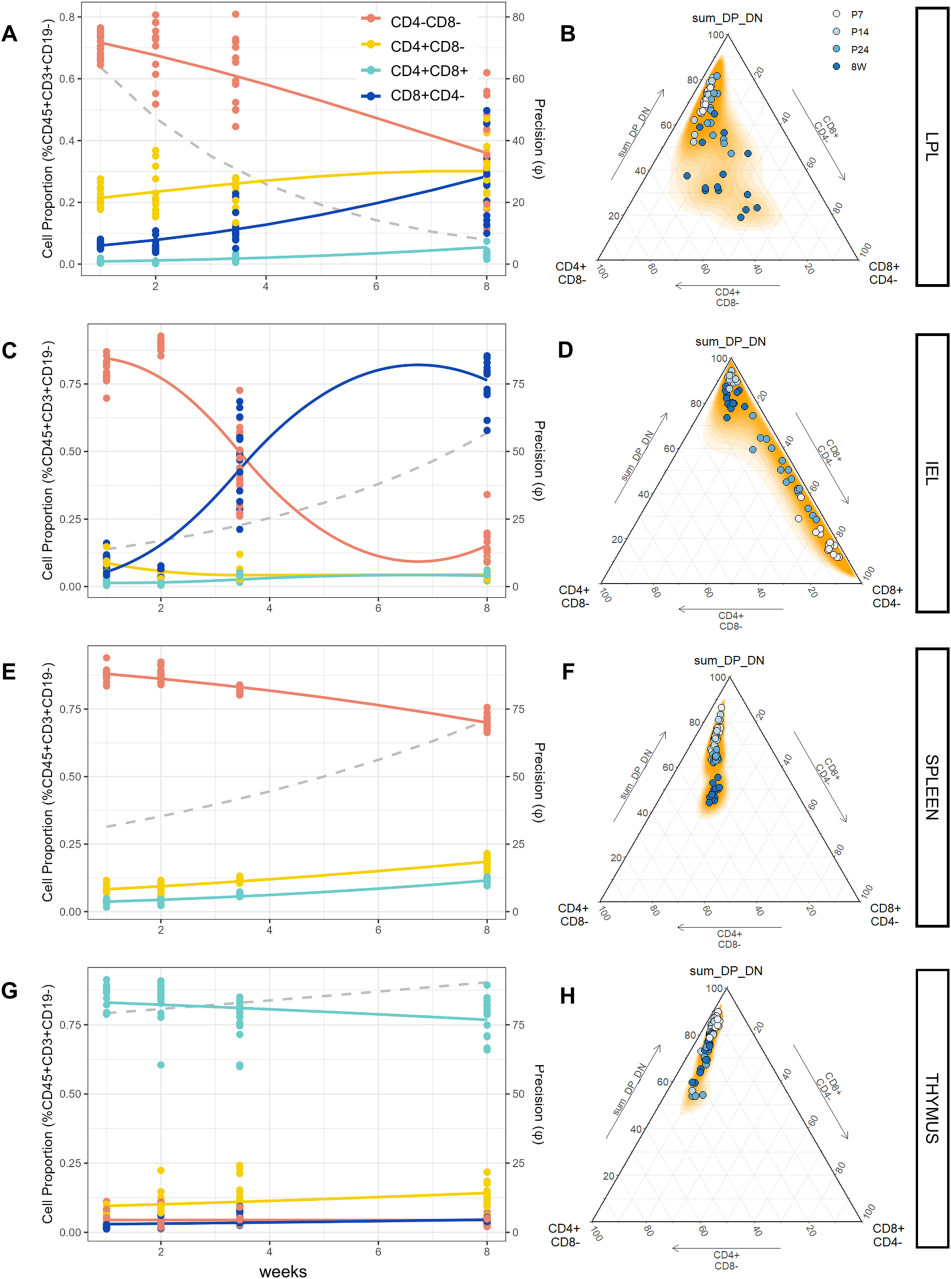
Overview of T-cell subsets across tissues over the postnatal period. **(A)** Intestinal Intraepithelial Lymphocyte (IEL), **(C)** Lamina Propria Lymphocyte (LPL), **(E)** Splenocyte (SPLN), and **(G)** Thymocyte (THY) populations expressing CD4/CD8 as a fraction of the live CD45+CD3+CD19- parent population. **(B)** (IEL), **(D)** (LPL), **(F)** (SPLN), and **(H)** (THY) show the corresponding ternary plot for each tissue, which maps these proportions to a compositional simplex to show relationships among variables whose values sum to a constant. Each triangle represents a sub-composition with three components: CD4+CD8- SP, CD8+CD4- SP, and a third (sum DP_DN) that is the sum of the proportions of the remaining two components (CD4-CD8- DN and CD4+CD8+ DP). The closer a point is to one of the corners, the higher the proportion of that component for that observation. Orange shading represents datapoint density.

The dynamics of T-cell populations within the total lymphocyte pool from each tissue is visualized in **Figure 2**. The left column of plots illustrates the trajectory of relative T-cell subset abundance as a percentage of CD45+CD3+CD19- cells; the right column shows corresponding ternary plots where each vertex represents CD4+ single positive (SP) cells, CD8+ SP cells, and combined abundance of CD4/CD8 double positive (DP) and double negative (DN) cells, respectively. In this context, proximity of a given point to a vertex indicates a high proportion of the population represented by that vertex in that sample. In alignment with previous results (53–55), gut mucosal immune populations showed significant variability across postnatal development, with significant changes occurring post-weaning (P24). Qualitatively, the modest increase in the proportion of LP CD3+ T-cells from P14 to P24 was driven by concomitant expansion of both CD4+ and CD8+ subgroups (**Figures 2A, B**). The robust increase in CD3+ T-cells in IELs between P14 and P24 was largely driven by an increased population of CD8+ lymphocytes (**Figures 2C, D**). According to regression results, a significant compositional difference in CD4/CD8 T-cell populations across time points was observed in both tissues (Λ_overall_ = 86.004 and 223.70 for IEL and LPL, respectively, p < 0.001). Univariate coefficients aligned with visually obvious sub-compositional shifts: in the LP, a significant increase in CD8+CD4- populations was observed between P14 and P24 (p < 0.001), and between P24 and 8W (p < 0.001). The latter shift was paralleled by a synchronized increase in CD4+ and CD4+CD8+ cells between P24 and 8W (both p < 0.001). In IELs, a significant positive shift in the proportion of CD8+ cells occurred between P14 and P24 as well as between P24 and 8W (p < 0.001). This was in parallel to a modest but significant increase in the proportion of DP cells across both age intervals (also p < 0.001).

In the spleen, there was a steady and coordinated increase in the abundance of CD8+ and CD4+ cells; by adulthood, the latter comprised roughly 20% of all CD45+ splenocytes (**Figures 2E, F**). As expected under normal conditions, DP CD4+CD8+ cells were not present (< 1%) (56, 57). By contrast, in the thymus the vast majority of CD45+ cells were DP, making up over 75% of total thymocytes with a slight decrease in abundance between P7 and adulthood (**Figures 2G, H**). This was mirrored by a modest increase in CD4+ cells across the lifespan in the spleen. Interestingly, the spleen displayed a significant increase in total T-cells (both SP CD4 and SP CD8) across the lifespan as a proportion of CD45+ cells, the *relative* proportion of CD4/CD8 populations did not change significantly over time (p = 0.137). This is illustrated in the ternary diagram shown in **Figure 2F**. Although there is a vertical shift away from the double negative (DN/DP) cell vertex, there is no perceptible shift on the horizontal axis, in contrast to LPLs and IELs which both showed significant changes across all axes (**Figures 2B, D, F**). In the thymus, regression results revealed no significant changes in the total proportion or relative composition of CD4/CD8 cells between time points, aside from a modest transition between P7 and P14 favoring SP CD4+ cells (**Figures 2G, H**, p < 0.05).

### 3.2 Development of functional T-helper cell subsets varied between tissues

**Figure 3:**
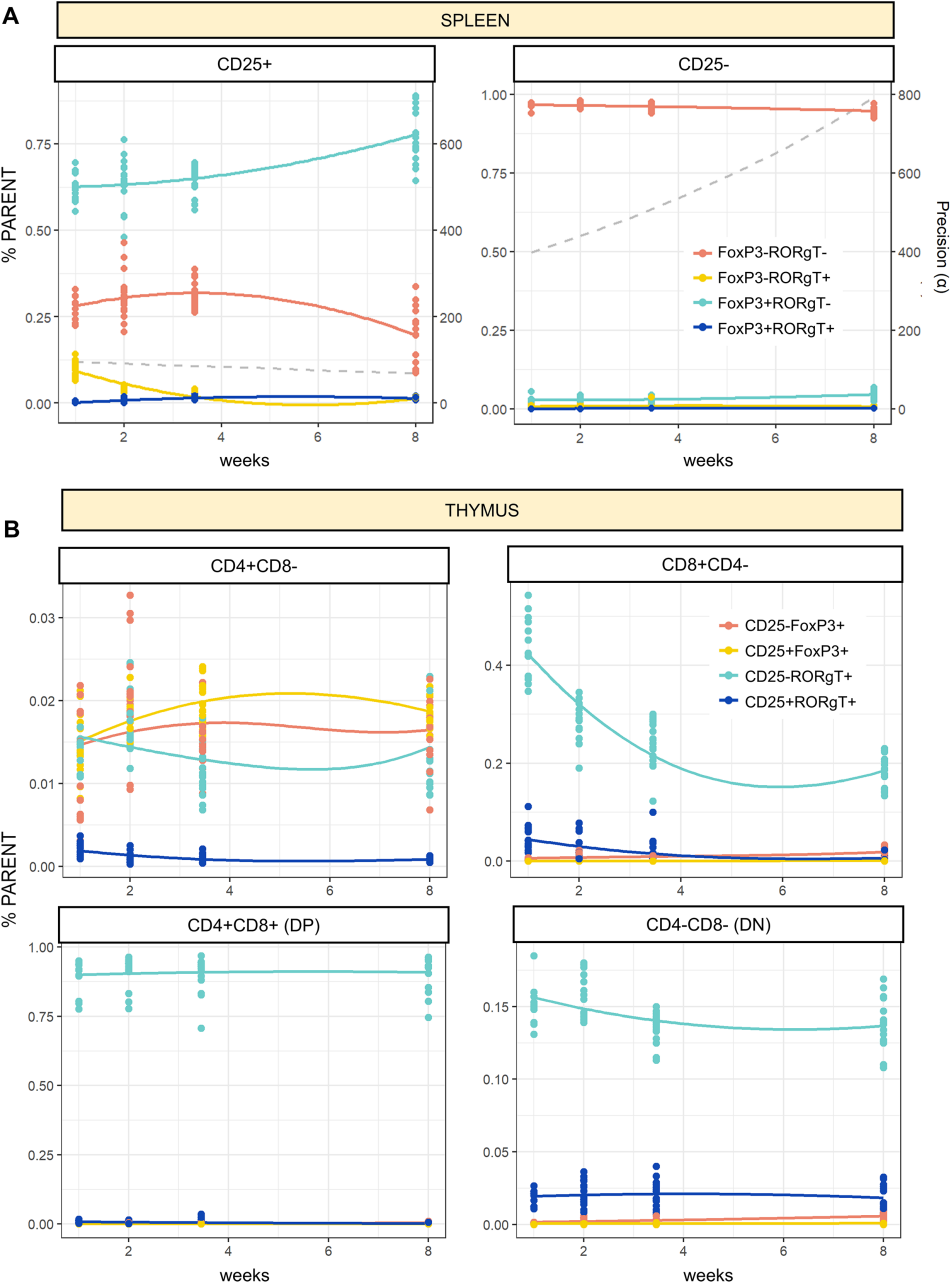
Overview of functional T-cell subsets in spleen and thymus tissues. **(A)** Proportions of RORγT/FoxP3 (+/-) cells in both CD25+ (left) and CD25- (right) parent gates. **(B)** Proportions of FoxP3/RORγT (+/-) cells in SP, DP and DN thymocyte populations. Smooth curves represent Dirichlet regression models fitted to weeks of age as a continuous variable for visualization purposes. Dashed lines represent precision (φ) values fitted from the same model.

Further resolving CD4+ Th cell heterogeneity, populations of T-regulatory (CD25+ FoxP3+, Treg) and Th17 (RORγT+) cells were mapped over time. As expected, the vast majority of CD25+ cells (60-75%) in the spleen were FoxP3+ Tregs and increased in abundance across timepoints (**Figure 3A**, left). Regression results showed a significant increase in the relative abundance of CD25+FoxP3+ splenocytes between P24 and 8W (p<0.001), with no differences observed between other timepoints (p = 0.231 and 0.876 for P7–14 and P14-24, respectively). A small but consistent population of CD25+ RORγT+ cells was observed at P7, but effectively disappeared by P24. Regression results confirmed a relative decline in this cell population within the first two age intervals (p<0.01), without further fluctuations beyond P24. Most cells in the CD4+CD25- splenocyte population we negative for either functional marker (FoxP3-RORγT-), but a small population of CD25-FoxP3+ cells (<5%) persisted from P7 to adulthood (**Figure 3A**, right).

In contrast to the spleen, all thymic T-cell populations aside from CD4+ SP cells were dominated by CD25-RORγT+ thymocytes. This was especially evident in the DP CD4+CD8+ population, which consistently expressed RORγT in >75% of cells across development (**Figure 3B**). CD8-CD4- DN cells also showed a modest but consistent population of RORγT+ cells (10-15%), while in CD8+ cells there was a relative decline in RORγT+ cell numbers over time to around 15-20% by 8W (**Figure 3B**). Although these functional markers have different implications at different stages of development and in different organs, these results illustrate a unique spatiotemporal pattern of T-cell populations across postnatal development, characterized by the proliferation and decline of different splenocyte and thymocyte subsets as the immune system matures.

**Figure 4:**
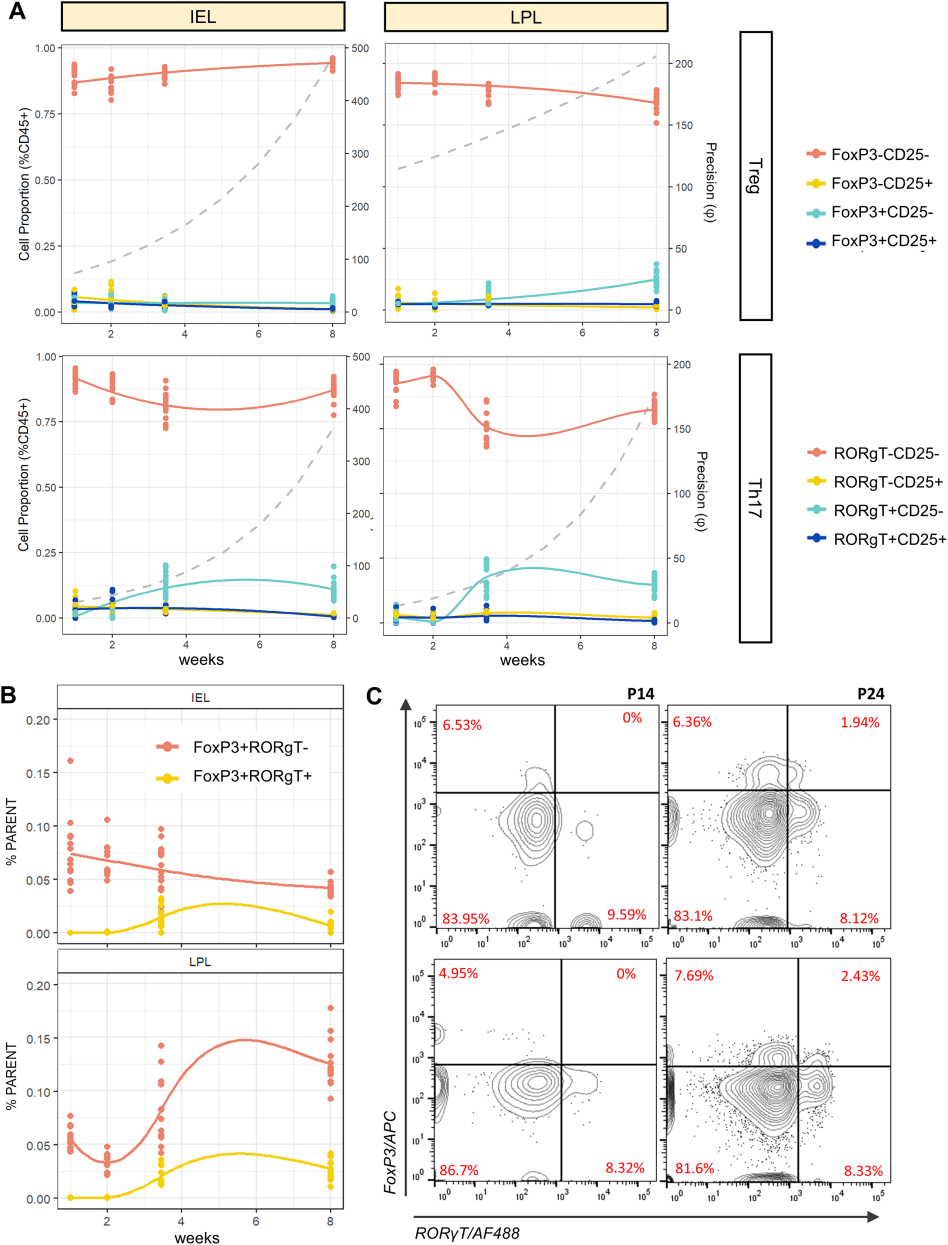
Overview of functional T-cell subsets in gut tissue (IEL and LPL) compartments. **(A)** Proportions of CD25(+/-)FoxP3(+/-) cells (including Tregs, top row) or CD25(+/-)RORγT(+/-) cells (including Th17 cells, bottom row) in both IEL (left) and LPL (right) populations. Smooth curves represent Dirichlet regression model fitted to weeks of age as a continuous variable for visualization purposes. Dashed lines represent precision (φ) values fitted from the same model. **(B)** Relative proportions of FoxP3/RORγT(+/-) pTreg cells measured as a proportion of CD4+CD8- T-cells. **(C)** Representative contour plots showing absence/presence pTregs at P14 (left column) and P24 (right column) in IEL (top row) and LPL (bottom row) compartments.

Intriguingly, a symmetry between IEL and LPL populations was observed in the levels of functional CD4+ T-helper cell populations. Both tissues showed a relatively low proportion of CD25+FoxP3+ Tregs that remained constant across time points (**Figure 4A**, top row). In the IEL, there was an uptick in the abundance of CD25+RORγT+ T-cells from P14-P24 which superseded CD25-RORγT+ cells, peaking at an abundance of ~7% at P14. In both tissues, there was then a large increase in abundance of CD25-RORγT+ cells between P14 and P24 (p<0.001), which then decreased slightly in both tissues by 8W (**Figure 4A**, bottom row). Interestingly, in the LPL there was an additional population of CD25-FoxP3+ cells that emerged around P24 and comprised >10% of Th cells by 8W. In addition to the canonical Tregs, the gut mucosa harbors a unique population of CD4+ peripheral regulatory T-cells (pTregs) expressing both FoxP3 and RORγT (58). Although this population makes up the minority of Tregs in the small intestine (SI), they play an important role in establishing immune tolerance during early life and weaning (59). In accordance with this, a small but notable population of RORγT+ pTregs appeared at P24 in both the IEL and LPL and persisted into adulthood (**Figures 4B, C**). These results suggest not only a dynamic evolution of intestinal T-cells in response to the environment but also demonstrated an apparent coordination between IELs and LPLs that could reflect immune system tuning by the microbiome.

In order to provide a comprehensive overview of T cell development, we also measured the relative proportions of αβ versus γδ T-cells across these immune compartments. As expected, in the spleen and thymus there was a near absence (<5%) of TCRβ- cells. In the IEL, by contrast, there was a clear separation of CD3+TCRβ+ and CD3+TCRβ- cells, which occurred in roughly equal proportions (45-55%) at P7 and 8W but were increased at P14 (**Supplementary Figure 5**). In the LPL, there was some variance in populations of TCRβ- cells (15-25%) as determined by FMO control. Both observations were borne out in beta regression results, whereby significant differences were observed between TCRβ+ populations between P7 and P14 in the IEL and LPL (p < 0.001), as well as between P14 and P24 in the IEL (p<0.001). However, additional tests showed no significant difference between P7 and 8W in either tissue (p = 0.282 and 0.216, respectively). This observation was confirmed directly in a follow-up experiment that measured TCRβ+ and TCRγδ+ cells directly at both P7 and 8W, which confirmed a roughly 85/15% split between these populations in the LPL and 50/50% in the IEL at both timepoints (**Supplementary Figure 5**).

### 3.3 Stepwise maturation of the microbiome was observed between pre- and post-weaning

**Figure 5:**
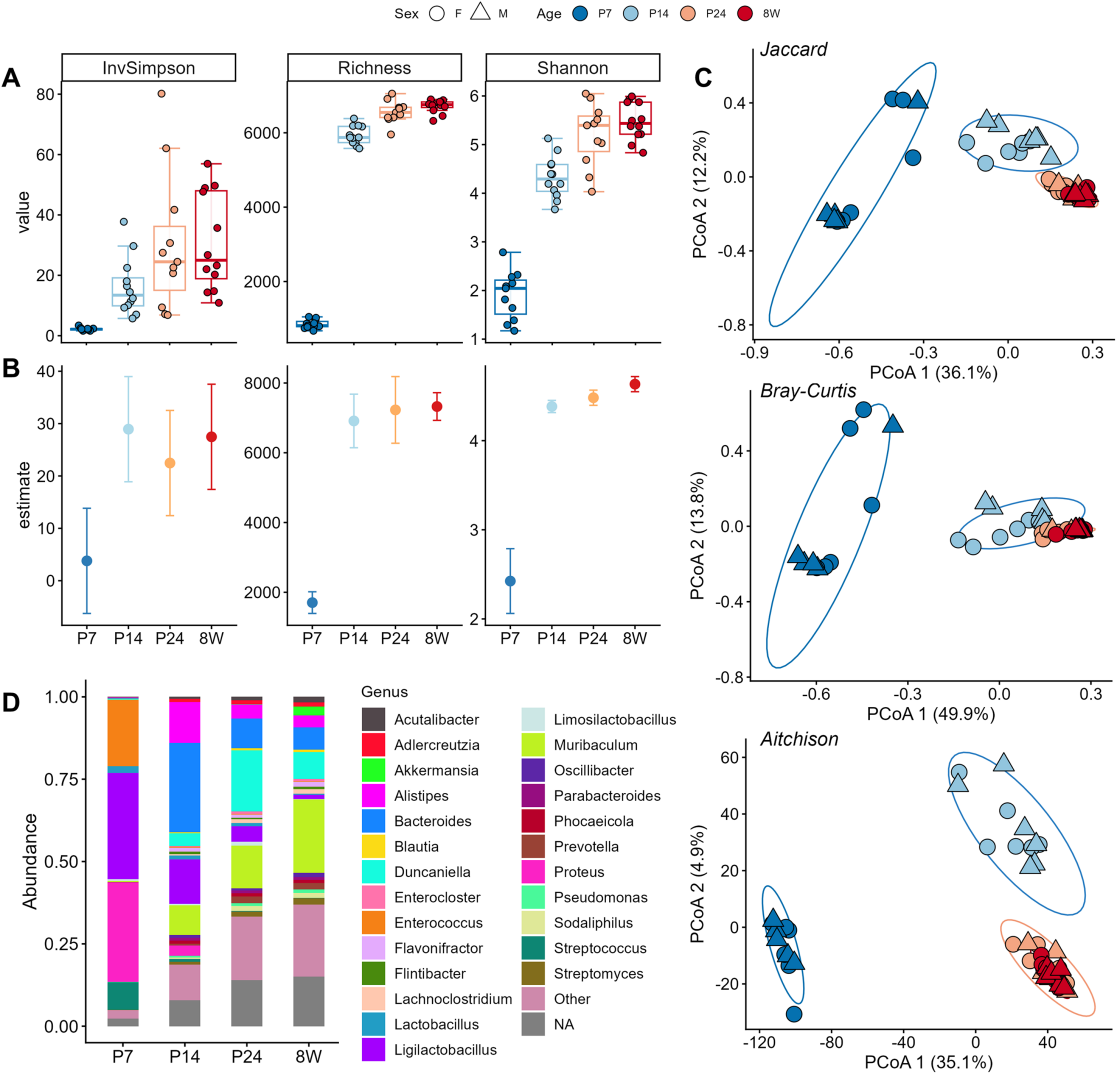
Developmental changes in microbiome diversity and composition. **(A)** IQR boxplots of alpha diversity measures across samples using the inverse Simpson index, total richness and Shannon index. **(B)** Alpha diversity measures (mean +/- SEM) estimated using the breakaway package. **(C)** PCoA decomposition of beta diversity distance matrices for Jaccard (top), Bray Curtis (middle) and Aitchison (bottom) distances. **(D)** Mean compositional bar plots of the top 25 most abundant retain-resolved genera across each timepoint group.

Shotgun metagenomic sequencing (MGS) showed that samples at P7 had the lowest total sequencing depth and the most heterogeneity compared to later time points, despite similar overall sample weights to P14 samples after pooling (data not shown). Rarefaction curves and alpha diversity analysis revealed that P7 samples also showed much lower levels of within-sample diversity and a lower number of observed taxonomic sequences, although sequencing depth in these samples did not appear to systematically impact microbiome composition according to beta diversity ordination plots (**Supplementary Figure 6A**). Nonetheless, we validated alpha diversity outcomes via both conventional metrics (Shannon, Inverse Simpson, and Richness) and data-driven estimates using the breakaway package (60) to account for potentially unobserved taxa in each sample group. Groupwise alpha diversity distributions are shown in **Figure 5A**; adjusted alpha diversity estimates are shown in **Figure 5B**. Statistical tests on original estimates found a significant effect of Age on all alpha diversity measures, and *post-hoc* tests confirmed a highly significant increase in diversity between P7 and all other time points (p<0.01), with an additional modest but significant increase between P14 and P24 in Shannon index and observed reads (p<0.05). Similar results were obtained when testing group differences in breakaway’s alpha diversity measures, which mostly aligned with our original results but provided slightly higher group-wise estimates across the board.

Beta diversity ordination plots revealed significant shifts in overall microbiota composition between P7 and P14 as well as between P14 and P24, with a high degree of overlap between clusters representing samples obtained at P24 and 8W (**Figure 5C**). Non-compositional beta-diversity measures showed high variability in the P7 samples compared to other groups (illustrated by a larger spread in the cluster), while Aitchison distance significantly reduced these irregularities in within-cluster distance (**Figure 5C** bottom panel), suggesting that compositional effects may strongly contribute to the variety of microbiome structures observed in early life. In alignment with these observations, PERMANOVA results suggested a significant main effect of Age on microbiome composition across all distance measures; pairwise comparisons revealed that this outcome was driven by significant differences between P7 and P14 as well as P24 (p<0.001), while the P24 versus 8W comparison did not reach significance (p=0.06 and 0.07 for Bray-Curtis and Aitchison distances, respectively). Together, these results indicate an interesting dynamic of microbiome diversification, whereby a highly variable but less diverse microbiome in early life rapidly diversifies by the second week and undergoes a significant compositional shift from pre-to post-weaning that quickly reaches, adult-like state at P24.

Prior to further downstream analysis, we implemented our retain-resolve (RR) method for denoising the data. Out of 7620 initial taxa, 268 met initial prevalence/abundance criteria and were retained in the analytical dataset. The remaining 7352 sequence-level taxa that did not meet criteria were agglomerated to 1892 genus-level taxa using the *tax_glom()* function. 207 glommed taxa met criteria in the second filtering round and were added to the analytical dataset. The remaining 1685 taxa were agglomerated into a single “other” category, resulting in a final dataset that included a total of 476 taxa. Compositional bar plots of retain-resolved taxa for the top 25 most abundant genera are shown in **Figure 5D**.

**Figure 6:**
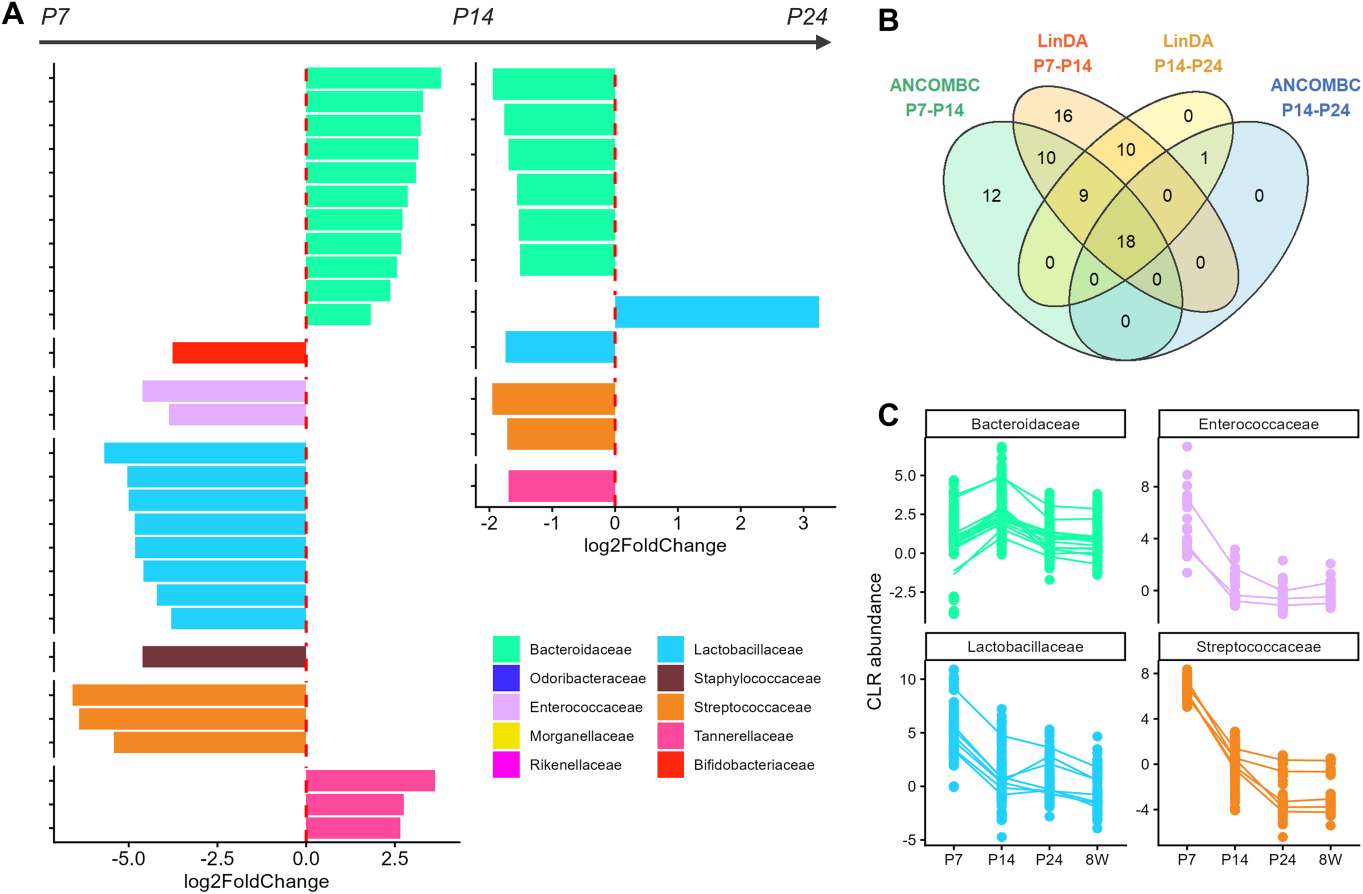
Summary of consensus differential abundance (cDA) analysis of the gut microbiome across development. **(A)** Log2fold change of consensus taxa between P7-P14 (left) and from P14-P24 (right). **(B)** Venn diagram of significant cDA taxa across both time intervals for LinDA (top ellipses) and ANCOM-BC (bottom ellipses) models. **(C)** Line plots showing changes in the CLR-transformed abundance of ASVs from most frequent cDA families across sampled timepoints.

To further explore which taxa are driving changes in microbiome composition across the lifespan, we performed consensus differential abundance analysis with a focus on which taxa changed significantly between adjacent time points (e.g. between P7 & P14, P14 & P24, and P24 & 8W). In alignment with earlier results, only one model (ANCOMBC) picked up on three taxa that were significantly different between P24 and 8W (*Limosilactobacillus reuteri, Ligilactobacillus murinus* and *animalis*) with a negative log2 fold-change (L2FC, between -2.8 and -3.2), indicating minimal changes in overall microbiome composition between P24 and adult mice. The majority of differentially abundant taxa in both models were observed between P7 and P14, with a consensus list of 30 RR-taxa from 13 different genera that showed significant changes in abundance between each time interval (**Figures 6A, B**). Several taxa belonged to the genus *Bacteroides*, all of which showed a large positive L2FC between P7 to P14 and a subsequent negative L2FC from P14 to P24. Other notable genera included *Streptococcus*, with a handful of associated taxa that decreased in relative abundance over the lifespan, and several genera in the family *Lactobacillaceae* (including *Lactobacillus*, *Ligilactobacillus* and *Limosilactobacillus)* that decreased in abundance during the second week of life. This aligns with individual compositional bar plots of the microbiota shown in **Supplementary Figure 5B**, and trends in mean CLR-transformed abundances of these taxa across timepoints as shown in **Figure 6C**. At P7 the microbiota is composed primarily of *Enterococcus*, *Ligilactobacillus* and *Proteus*, which are superseded by *Bacteroides* at P14. Subsequently, *Bacteroides* was supplanted by *Muribaculum* and *Duncaniella* as the predominant genera by P24. Despite the average decrease in *Proteus* abundance from the first to the second week of life, individual bar plots revealed that this was largely driven by 3 samples whose microbiota comprised >60% *Proteus* taxa, and thus the within-group variance likely masked any mean differences.

### 3.4 Integration of microbiome and T-cell population data

**Figure 7:**
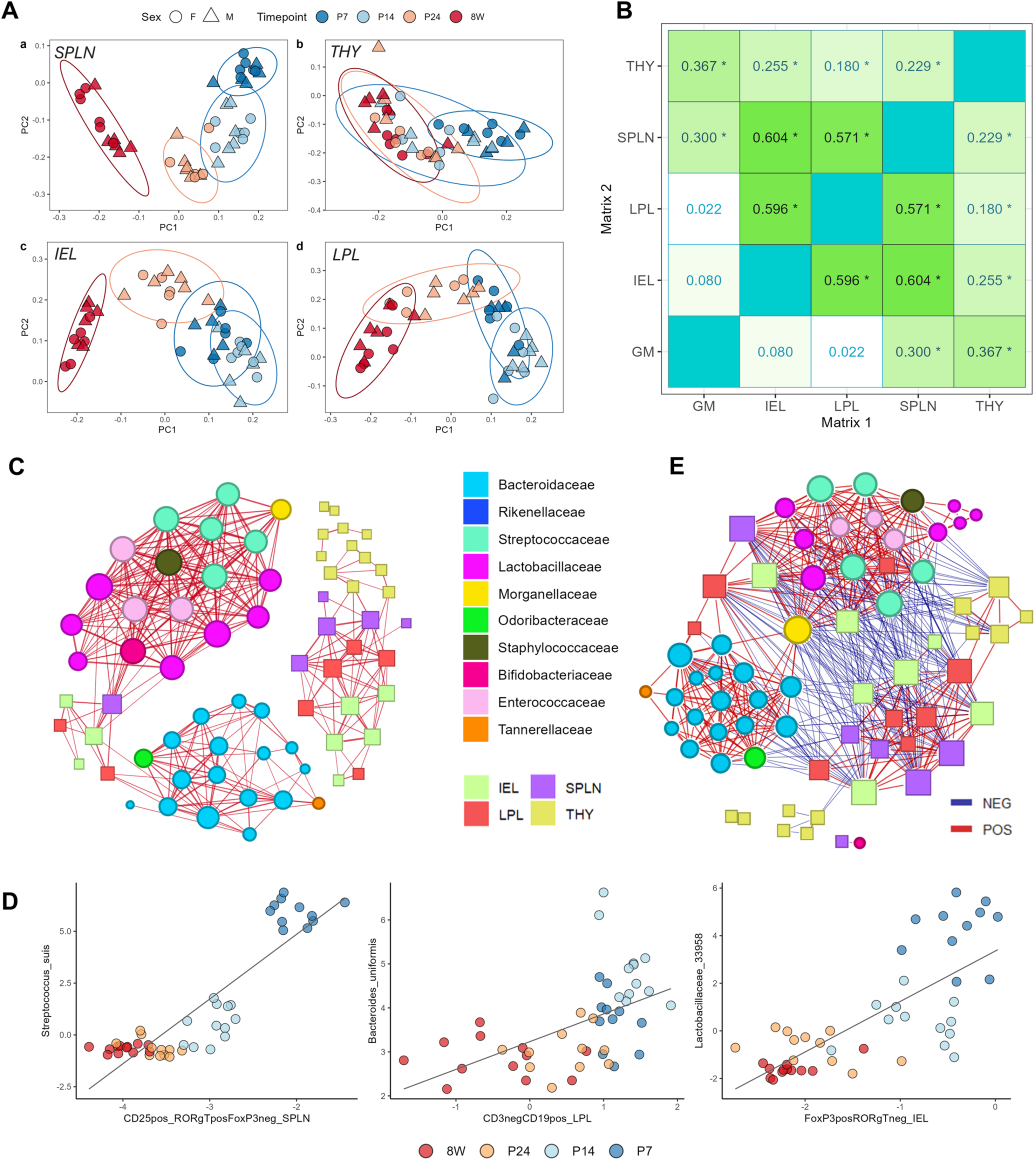
Integrated microbe-immune analysis. **(A)** PCA decomposition of unzeroed, stabilized and standardized (USS) immune cell data for the spleen (upper left), thymus (upper right), IEL (bottom left) and LPL (bottom right) compartments. **(B)** Mantel analysis correlation matrix for all immune cell-microbiome distance matrix pairs; values significant to an FDR < 0.05 are indicated with an asterisk (*). **(C)** Network plot displaying all significant proportionality measures (rho > 0.45) between consensus ASVs (circles) and immune cell subsets (squares). **(D)** Spearman correlation network constructed using CLR-transformed microbiome and immune cell data. **(E)** Scatter plots of exemplary associations between CLR-transformed abundance of consensus taxa and USS immune cell subset data.

In order to integrate microbiome and flow cytometry data, we applied an unzeroing, stabilization and standardization procedure on immune cell abundance tables to transform hierarchical percentage data into multivariate compositional space (**Figure 7A**). To explore the global relationship between microbiome and flow cytometry data, we next conducted a Mantel test on the microbiome (Aitchison distance) and immune cell distance matrices (Euclidean distances calculated on log-ratio transformed data, **Figure 7B**). We found that all immune cell datasets were highly correlated, indicating a significant relationship between the covariance of different immune cell subtypes. Interestingly, the strongest relationship was observed between the IEL and SPLN distance matrices (r = 0.604), followed by the association between IEL and LPL (r = 0.596), and SPLN and LPL (r = 0.571) immune cell compositions, suggesting that mucosal immune cells strongly covary with splenocyte populations. Furthermore, the gut microbiome distance matrix (GM) was only significantly correlated with splenocyte and thymocyte compositions (r = 0.28 and 0.37, respectively, p<0.05) but neither lymphocyte population matrix in the gut (r = 0.057 and -0.04, NS), suggesting a stronger relationship between distance matrices representing gut microbial ecology and primary immune organ lymphocyte populations.

Intrigued by this result, we next performed integrated Compositional Data Analysis (CoDA)-adapted “correlation” analysis to determine specific immune-cell microbe associations impacted by developmental stage. This was achieved using the propr R package, which measures the proportionality of pairs of variables following log-ratio transformation (see Methods). We applied this method to CLR-transformed immune cell data from each tissue and the subset of 53 consensus differentially abundant taxa significantly associated with transitions between postnatal timepoints (**Supplementary Table 3**). Using a cutoff of 0.45 for the proportionality constant (ρ) we extracted all significant associations between immune cell populations and microbial taxa (corresponding to an FDR < 0.05) and constructed a multi-modal feature network shown in **Figure 7C**. The developmentally-regulated taxa formed two dense proportionality networks: one dominated by *Bacteroides* taxa, and the other comprised mostly of microbes in the *Lactobacillaceae* and *Streptococcaceae* families. These dense modules were connected by a relatively sparse network of mixed-origin immune cells. This intermediate immune cell group included CD25+RORγT+ T-cells in the spleen, CD25+FoxP3+ IELs, and LP B-cells (**Figure 7C** and **Table 1**). More specifically, we observed that LPL B-cells were associated with three strains of *Bacteroides* (*B. uniformis*, *spA1C1* and one unidentified strain), while CD19+ B-cells in the spleen and CD4+ LPLs were uniquely connected to a single *Muribaculum* (*M. intestinale*) associated with the *Bacteroides*-dominated cluster. FoxP3+ IELs were strongly associated with various taxa from the genera *Lactobacillus*, *Enterococcus*, *Staphylococcus*, *Bacteroides*, and *Bifidobacterium*. CD25+RORγT+ cells in the spleen were also associated with microbial taxa from these genera, in addition to strong links with two *Streptococcus* taxa (*S. suis* and one unidentified). Some of these key microbe-immune cell associations are illustrated in **Figure 7D**.

**Table 1:** Significant proportionality relationships (rho > 0.45, corresponding to FDR < 0.05) between ASVs and immune cells.

Pair name	Partner name	rho
CD25pos_RORgTposFoxP3neg_SPLN	*Streptococcaceae_Streptococcus_suis*	0.475
CD25pos_RORgTposFoxP3neg_SPLN	*Streptococcaceae_Streptococcus_NA_1328*	0.467
FoxP3posRORgTneg_IEL	*Staphylococcaceae_Staphylococcus_NA_1279*	0.453
CD25pos_RORgTposFoxP3neg_SPLN	*Staphylococcaceae_Staphylococcus_NA_1279*	0.554
CD4posCD8neg_LPL	*Muribaculaceae_Muribaculum_intestinale*	0.465
CD3negCD19pos_SPLN	*Muribaculaceae_Muribaculum_intestinale*	0.573
FoxP3posRORgTneg_IEL	*Lactobacillaceae_Lactobacillus_NA_1584*	0.510
CD25pos_RORgTposFoxP3neg_SPLN	*Lactobacillaceae_Lactobacillus_NA_1584*	0.602
CD25pos_RORgTposFoxP3neg_SPLN	*Enterococcaceae_Enterococcus_NA_1350*	0.523
FoxP3posRORgTneg_IEL	*Enterococcaceae_Enterococcus_NA_1352*	0.494
CD25pos_RORgTposFoxP3neg_SPLN	*Enterococcaceae_Enterococcus_NA_1352*	0.583
FoxP3posRORgTneg_IEL	*Bifidobacteriaceae_Bifidobacterium_NA_216816*	0.504
CD25pos_RORgTposFoxP3neg_SPLN	*Bifidobacteriaceae_Bifidobacterium_NA_216816*	0.606
CD3negCD19pos_LPL	*Bacteroidaceae_Bacteroides_spA1C1*	0.478
FoxP3posRORgTneg_IEL	*Bacteroidaceae_Bacteroides_NA_816*	0.460
CD3negCD19pos_LPL	*Bacteroidaceae_Bacteroides_NA_816*	0.527
CD3negCD19pos_LPL	*Bacteroidaceae_Bacteroides_uniformis*	0.491
FoxP3posRORgTneg_IEL	*Lactobacillaceae_NA_NA_33958*	0.466
CD25pos_RORgTposFoxP3neg_SPLN	*Lactobacillaceae_NA_NA_33958*	0.537

To further understand the temporal patterns driving these omnibus immune-microbiome network interactions, we performed the same network construction procedure (propr with 999 permutations, ρ >0.45) for each timepoint (P7, P14, P24, 8W) independently (**Supplementary Figure 7**). Results illustrate a progressive evolution of network structure from P7 to adulthood, wherein the same Bacteroides-dominated cluster begins to emerge by P14 and is entirely distinct from the surrounding network at 8W. Other than this singular cluster, timepoint-specific networks did not reflect the same network structure observed in the omnibus analysis, but showed many additional microbe-immune cell associations. Another key difference was the absence of a second *Lactobacillaceae* and *Streptococcaceae*-dominated cluster (as observed in the omnibus network) from all timepoint-specific networks, suggesting that this cluster might be comprised of microbe-microbe associations emerging over developmental time as opposed to a modular structure that remains consistently present across multiple timepoints.

To account for the fact that proportionality does not provide a reliable indication of inverse relationships (analogous to negative correlations), we constructed a bootstrapped Spearman correlation network on CLR-transformed immune cells and differentially abundant bacterial taxa. Strikingly, this method yielded an association network with many similarities to the proportionality network, including the presence of two densely connected modules dominated by Bacteroides and *Lactobacillaceae*/*Streptococcaceae* taxa, respectively (**Figure 7E**). Immune cell populations in the spleen, IEL, and LPL compartments were integrated with microbial clusters, showing stronger links to *Lactobacillaceae, Enterococcaceae*, and *Streptococcaceae* taxa in addition to forming links with the *Bacteroides* cluster via LPL CD19+ B-cells and FoxP3+RORgT- Th cells. Notably, inclusion of negative associations revealed structural antagonism between bacterial modules in the network, as well as significant negative relations between bacteria in both clusters and immune cell subsets, suggesting the suppression of some bacterial communities by immune regulators.

To confirm the significance of the observed global network structure, we implemented a random matrix theory (RMT) approach to compare observed networks with randomly permuted networks of equal size and average degree. In global tests, both networks displayed significantly higher modularity and clustering coefficients than their randomly permuted counterparts, as well as significant differences in diameter, average path length (APL) and centrality (**Table 2**). Additionally, bacterial clusters showed a high degree of stability across bootstrapped iterations in both network types (data not shown), further supporting the existence of non-random network interactions between microbial consortia and gut immune cells. Finally, hierarchical clustering was performed to assess the stability and modularity of global networks. As seen in **Supplementary Figure 8**, results of this unsupervised method support the above-described network structure, wherein three main clusters were observed: two dense bacterial clusters that were interconnected by sparsely associated immune cell populations, and a relatively isolated cluster of immune cell subsets. Combined with previous results, this finding further supports the intermediate role of immune cells in coordinating developmental interactions between key microbial consortia.

**Table 2:** Network topology measures of empirical and randomly generated networks for propr (proportionality) and spearman-CLR methods.

Measure	Propr		Spear-CLR	
	Observed	Random	Observed	Random
*Nodes*	69		68	
*Edges*	313		518	
*Density*	0.133		0.227	
*AD*	9.07		15.23	
*ACC*	0.797*	0.133	0.677*	0.226
*APL*	1.396*	2.12	1.52*	1.795
*Diameter*	2.78*	3.88	3.64*	3.01
*Modularity*	0.62*	0.25	0.352*	0.169
*Btw. Centrality*	23.9*	38.312	38.18*	26.64

AD, average degree; ACC, average clustering coefficient; APL, average path length.

*Significant difference (rand. vs. obs.) of p<0.01.

## 4 Discussion

Colonization and maturation of the gut microbiome during postnatal development influences the concomitant development of the adaptive immune system. Previous work has demonstrated that cross-talk between gut microbiota and T-cells during this postnatal window influences brain development and behavior (18, 19, 61, 62). Using a robust compositional framework to phenotype multi-organ adaptive immune and microbiome signatures throughout development, the current work mapped T-cell ontogeny in mucosal and systemic immune compartments to gut microbial maturation during postnatal development. Not only did this approach provide insights beyond the scope of traditional analytical tools, but defined a roadmap for co-development of lymphocyte populations and gut microbiome communities in both male and female mice under healthy conditions. These data show that the microbiome and adaptive immune system follow coordinated but distinct patterns of compartment-specific development in the pre-weaning period, whereby sequential waves of dominant gut taxa were accompanied by expansion and stabilization of functional and regulatory T-cell subsets. Furthermore, vertical integration of this data identified a modular network of associations between functional T-cell subsets and densely connected communities of gut bacteria.

The developmental trajectory of T-cell subsets over the first 3 weeks of postnatal development distinct in peripheral and mucosal immune compartments. T-cell development in the thymus begins mid-gestation where bone marrow progenitors enter the thymus and generate thymocytes. These cells, initially DN CD4-CD8-, develop into DP CD4+CD8+ thymocytes that then proliferate and differentiate into mature functional T cells and are subsequently exported from the thymus (63). During postnatal development, immature (DN) thymocytes are abundant and undergo proliferation and differentiation (63). Our data mirror these trajectories but also revealed some interesting developmental patterns in functional marker abundance, although it should be noted that these thymic transcription factors have different implications than in other peripheral tissues. In contrast to the periphery, where expression of RORγT is interpreted as a hallmark of pro-inflammatory Th17 cells, in the thymus, it is essential for the survival of DP thymocytes. Moreover, downregulation of RORγT allows the maturation of CD4 SP and CD8 SP thymocytes (64, 65). This would explain the large fraction of RORγT+ cells in DP thymocytes (80-95%) which aligns with previous measurements (66, 67). The decline in total thymocyte populations in adult mice aligns with thymic involution that begins around 4–6 weeks of age (68). The results shows that the relative proportion of RORγT+ DP cells remained largely constant across the lifespan and despite the existing data to suggest that RORγT expression is restricted to DP thymocytes, we also observed a significant proportion of CD8 SP and DN cells expressing RORγT, which did not disappear upon adjustment of the CD4/CD8 gate to include more cells in the DP quadrant (data not shown). There are a few possible explanations for this observation. For one, RORγT expression was reported to be high when immature single-positive CD8+ cells differentiate into CD4+ CD8+ DP thymocytes, therefore this observation may be capturing a transitional population (69, 70). This would align with the observation that the SP CD8+ RORγT+ population declined from ~40% at P7 to <20% in adults, given the known decline of thymic output and T-cell flux across time. Furthermore, previous observations have shown that RORγT expression is present in the DN stage, giving rise to a unique lineage of invariant natural killer T (iNKT) cells. Rather than arise from DP precursors, these cells maintain RORγT expression during the maturation process and have a CD4- surface phenotype (71, 72). Other reports have further supported the presence of a population of RORγT+ IL-17-producing thymocytes that are not CD4+ T cells (i.e., classical Th17 cells), but comprise a heterogeneous population of CD8+, DN, and DP cells (73). Thus, the precise identity of these RORγT+ thymocyte populations remains unclear without additional surface marker analysis. Our results demonstrate that these cells are present in detectable levels under a healthy developmental context and might represent transitional populations or an unconventional iNKT lineage following a unique developmental program in the thymus.

In depth profiling of immune compartments over the postnatal period demonstrated differences between T-cell population dynamics in mucosal immune compartments (IELs and LPLs) compared to primary immune organs. By comparison, IEL and LPL populations showed large increases in the abundance of CD8+ T cells across the lifespan as well as unique proliferation patterns of functional surface marker expression, including a ~10% increase in the abundance of RORγT+ cells in both lymphocyte populations. These results empirically support the notion that, given their exposure to a highly dynamic luminal environment, mucosal immune cells would show larger variation in their functional and compositional makeup as the organism matures and highlight the importance of understanding how microbiota-immune crosstalk may influence these key developmental milestones in adaptive immunity.

In addition to functional subsets, another key variable of interest in the mucosa was the relative proportion of TCRβ and γδ T-cells. γδ T-cells follow a unique developmental program compared to αβ T-cells: while the latter progress through the canonical DN, DP and SP stages in the thymus, γδ T-cells deviate from this lineage at the DN stage and are mainly educated in the periphery (74, 75). Despite their rare occurrence in most tissues (<5% of total T cells), γδ T-cells comprise up to 40% - 60% of all IELs and ~15% of LPLs in adult mice (55, 76). This intestinal lymphocyte subgroup plays an essential role in surveilling the luminal environment, maintaining intestinal homeostasis, and suppressing microbial populations (77). In alignment with previous results, we observed a large proportion of LPLs expressing TCRβ, which peaked at ~82% at P14. A similar pattern was observed in the IEL, where the proportion of TCRβ+ cells peaked at ~65% at P14. A previous study on murine IELs showed similar results, although the researchers only sampled mice from 2–9 weeks of age (53). Curiously, based on the peak in abundance of TCRβ+ cells at P14, they extrapolated this trend backwards in time to conclude that the neonate gut must contain mostly TCRβ+ IELs and was colonized by γδ cells later in development. However, we observed a higher abundance of TCRβ-/TCRγδ+ IELs at P7, indicating that waves of γδ T-cell migration begin within the first week of life. This aligns with more recent studies demonstrating that IELs expressing the Vγ7 TCRγ chain segment are readily detectable as soon as 2 days after birth, reaching a plateau of 60–70% of the total CD3+TCRβ− IEL population by day 4 (78). Together with our previous findings, this result shows that the neonate mucosal immune system is highly dynamic and may display important developmental processes within the first postnatal week that warrant more thorough characterization.

The gut microbiota undergoes a dynamic developmental trajectory in response to host factors and the environment. Consistent with other reports, a dramatic shift in microbiome composition was evident between the first and second week of life, as well as from pre- to post-weaning. This was reflected in several differentially abundant taxa when comparing the microbiota of these sequential time points. By contrast, we observed few additional changes in microbiota composition and no significant differentially abundant taxa between P24 and adult, suggesting that the microbiome has already reached a mature, steady state just a few days after the weaning transition. This aligns with previous observations wherein the microbiome of weaned mice showed high overlap with adults as early as four days post-weaning (79). Importantly, the same study also measured the colonic microbiota at P7 and P14 and showed very similar results to the present experiment regarding within-timepoint variability, large compositional shifts, and increases in richness/alpha diversity over time. Indeed, the functional and taxonomic profile is known to be very unique pre-weaning, a period during which nourishment is restricted to maternal milk and bacterial communities undergo large fluctuations as they establish a niche in response to changing environmental conditions (80, 81). In this study, many fluctuations were observed in key bacterial taxa during this early window. For one, between P7 and P14, there was a notable increase in the abundance of numerous *Bacteroides* taxa, concomitant with decreases in the abundance of various *Lactobacillaceae* genera and *Streptococcus* taxa. Between P14 and P24, *Duncaniella* and *Muribaculum* taxa supplanted *Bacteroides* and *Alistipes* as the dominant genera. In alignment with beta diversity results, our consensus method detected no significant changes to retain-resolved taxa between P24 and adulthood. Indeed, our data suggests that microbiome development occurs in successive waves, whereby dominant taxa proliferate and then are subsequently overtaken by new consortia that acquire a competitive advantage in the intestinal ecosystem (80). Our data aligns with previous reports showing that the neonate mouse microbiota is dominated by *Lactobacillus*, which in both mice and humans delivered by natural birth is largely reflective of the mother’s vaginal microbiome (82–85). This is reportedly followed by a subsequent expansion of the *Bacteroides* genus and an increase in alpha diversity into the second week of life (82, 83, 86), as was observed in this experiment. Finally, the observed expansion of *Duncaniella* and *Muribaculum* from pre-to post-weaning is exemplary of a shift toward a mature microbiome composition; both these genera belong to the *Muribaculaceae* family, a functionally distinct group of carbohydrate-utilizing that dominates (>30% relative abundance) the adult mouse microbiota (87). We were, however, unable to find a logical cause for the high degree of variability in the P7 microbiota samples, whereby some profiles were dominated by the genus *Proteus* or *Enterococcus* and had reduced or insignificant abundance of *Lactobacillus*. Although the variability of the neonate microbiota is well known (80), and these genera have been previously reported at high levels in neonates (88, 89). Future research should further investigate the mechanisms of this differential initial colonization and how it relates to future health outcomes.

In addition to driving succession of microbial communities, the weaning transition plays an important role in the development of the immune system. This microbiome-dependent “weaning reaction” produces a spike in the pro-inflammatory cytokines tumor necrosis factor-alpha (TNF-α) and interferon-gamma (IFNγ) in the gut, triggering proliferation of a gut-resident population of Tregs (90, 91). Suppression of the weaning reaction leads to lifelong defects in the regulation of intestinal inflammation and gut barrier dysfunction, highlighting the importance of the early-life microbiota for immune system education and long-term immune homeostasis (86, 90). Thus, it makes logical sense that the observed shift in microbiome composition during this transition period would be paralleled by large shifts in gut-resident IEL and LPL T-cell subsets, as was observed in the data presented here. To further explore this finding, we referred to studies showing that the gut harbors a unique population of CD4+Foxp3+RORγ+ peripheral T-cells (pTregs) that are induced by specific commensal microbes and food antigens during weaning (92, 93). Our results confirmed the proliferation of nominal pTregs at P24 and adulthood (1.5-5% of total CD4+ cells, IEL and LPL) while this population was effectively absent at P7 and P14 (<0.1%). Furthermore, there was an additional FoxP3-RORγT+ cell population that dramatically increased from P14 to P24, which is consistent with previous reports that the appearance of Th17 cells in the LP correlated with the colonization of the intestines with normal commensal bacteria upon weaning (94). Taken together, our results accurately reflect the complex dynamics of intestinal T-cell subsets in the gut previously observed in multiple studies, in alignment with the dynamic response of the gut microbiome to the post-weaning environment.

The mechanisms connecting differentiation of T-cell subsets to specific microbial communities have largely been studied in the context of a mature organism and gnotobiotic conditions (reviewed in Shim, Ryu (95)). Our results revealed dramatic shifts in both microbial composition and immune cell populations in the first two weeks of life and between P14 and P24 (post-weaning). Notably, we also observed some important changes in immune cell composition, especially in the mucosal compartments, after the gut ecosystem converged to a mature, steady-state composition. The apparent “uncoupling” may also help to explain the relatively weak correlations between the gut microbiome and immune cell distance matrices (ex. GM vs LPL) compared to the strong associations between those calculated on immune cells from different organs (ex. SPLN vs. IEL). This observation suggests a “critical window” in early development during which microbiome-immune interactions play a dominant role in shaping certain aspects of host immunity.

Indeed, several studies have highlighted this concept using germ-free (GF) and immune deficient mouse models (19, 96, 97), whereby microbiome or immune manipulations during circumscribed windows can have outsized impacts on the developmental trajectory of certain phenotypic characteristics. For one, it is well known absence of gut microbes stunts the proliferation and activity of immune cells; GF mice present innate and adaptive immune deficits in adulthood. Although many of these abnormalities can be corrected via microbiome transfer at any age, a short GF postnatal period is sufficient to permanently alter certain aspects of host immune phenotype (98, 99). This includes not only the systemic levels of regulatory T cells, NK and NKT cells (98), cytokine production (98), and T-cell dependent regulation of IgE (100), but also the transcriptional profile of innate defense genes expressed by gut-associated immune tissue (101). In the opposite paradigm, immune deficiency has a marked impact on microbiome composition and functional output (26). Indeed, it was shown that genetic depletion of the adaptive immune system (*Rag1-/-* mice) leads to a post-weaning explosion in microbiome diversity, which may be related to a lack of regulatory feedback by T and B-cells (102). Furthermore, the microbiome of immune-deficient models is functionally and compositionally distinct, while restoring the immune system via adoptive naïve T-cell transfer or bone marrow transplant has marked effects on microbiome composition, and a suppressing effect on post-weaning diversity (25, 102). Interestingly, this result can be achieved even upon immune reconstitution in adulthood, suggesting that there is a certain degree of plasticity in microbe-immune interactions that persists into maturity. However, it should be noted that naïve CD4+ T-cell transfer also serves as an established model for inducing experimental colitis- that is, the post-reconstitution regulation of the microbiota comes at the cost of a significant, pathological pro-inflammatory response (103, 104). Thus, it could be theorized that the “critical window” concept may not represent a strict timeline for the restoration of immune phenotypes per se, but rather a period in which the immune system is biased toward preferential tolerance of stimuli (105) and thus forms the basis for homeostatic microbiome-immune interactions in adulthood. Further experiments should explore the temporal relevance of adaptive immune signals, in addition to the mechanisms that distinguish microbial tolerance from inflammation upon T-cell introduction.

In accordance with global results, associations between specific immune cell subsets and microbial taxa appeared to be driven by age-associated changes in relative abundance during early life. In the proportionality network, it was clear that temporal dynamics of specific immune cell subsets in early life (P7 and P14) contributed more to observed association with network taxa whereas little variation in either population was observed beyond weaning. Within the network was a clear separation between two main “modules” of differentially abundant bacteria, one dominated by *Bacteroides* strains and the other containing a mixture of taxa from the *Streptococcaceae*, *Enterococcaceae*, and *Lactobacillaceae* families. These densely connected modules were, in turn, connected by a sparse network of immune cells from the gut and spleen. B-cells in the LP and spleen were associated with the dense network of *Bacteroides* taxa (including *Muribaculum intestinale*), while functional Th cells (FoxP3+ IELs and RORγT+ splenocytes) were associated with a second heterogenous cluster of taxa from the genera *Lactobacillus*, *Enterococcus*, *Staphylococcus*, *Streptococcus*, *Bacteroides*, and *Bifidobacterium*. Notably, bacterial network modules aligned with results of the previous cDA analysis, where coordinated fluctuations in these genera were the largest contributors to age-related changes in microbiome composition between P7, P14 and post-weaning. Additional mechanistic literature has also demonstrated the links between some of these taxa and mucosal immunity, including increased production of IgA via T-cell-dependent B-cell activation pathways in both the small and large intestine (106–108), and proliferation of CD8+ IELs from CD4+ Treg precursors in the LP (109, 110).

In addition to omnibus results, further examination of timepoint-specific multi-omic networks revealed that some microbe-immune and microbe-microbe associations emerged from linearly distributed clusters of feature abundance corresponding to each age category, while others recurred as within-group associations across multiple timepoints. This raises the intriguing idea that some microbe-immune interactions follow a temporal gradient, while others persist in age-specific network dynamics. However, further research with larger group-wise sample sizes should explore the presence of differential microbe-immune co-abundance networks between each developmental stage and map these relationships to dominant mechanisms of microbe-immune interactions over time.

This analysis implemented a novel combination of compositional techniques applied to both independent and integrated immune-microbiome analysis, illustrating the utility of these principles in flow cytometry to examine changes in immune cell populations between groups. This was achieved using a combination of data transformation and a statistical approach that accounted for compositional and hierarchical properties of the data, maximizing robustness of results and interpretability of multi-omic integration. As described in the methods section, applying standard statistical tools to such data is problematic and suffers from many of the same pitfalls as treating microbiome relative abundance data with these techniques. Compositional data analysis (CoDA) has recently shown a huge increase in popularity across multiple fields, implementing specific mathematical transformations and statistical frameworks to address the inherent mutual dependency between data components and compositional bias. The advantages of CoDA have been thoroughly reviewed in a variety of contexts (111), however these techniques remain slow to catch on in many disciplines. Indeed, very few flow cytometry studies implement robust compositional analysis, and the “norm” in the field remains parametric statistical tools that may not accurately model the data (38). In this case, we first performed compositionally sound analyses on both independent microbiome (Aitchison distance, LinDA, ANCOM-BC) and immune datasets (Dirichlet regression), which not only produced interesting conclusions regarding the developmental dynamics of these systems but guided our hypotheses when designing an integrated framework. The latter analysis relied on distance-based analysis (Mantel tests) using metrics that accounts for the relative scale property of compositional data (Aitchison distance), as well as proportionality analysis in lieu of standard correlation network analysis to reduce spurious relationships between vertically integrated data features. There were some key places where the importance of this approach was emphasized, especially with respect to the framing and presentation of conclusions. As an illustrative example, spleen data showed an incremental increase in the abundance of CD4+ and CD8+ cells over time; univariate tests and standard regression would likely conclude that these increases were independently significant and report them as such. However, Dirichlet regression tells a different story: while the relative abundance of both these cell type does increase, their relative proportions as a fraction of total T-cells do not. Thus, the increase is likely due to an increase in total T-cell abundance in the spleen, thereby increasing the abundance of each constituent subset due to their negative correlation with DN cells. Although this may seem like a minor difference, depending on the context these conclusions may lead to entirely difference follow-up strategies exploring biologically relevant mechanisms. Furthermore, while proportionality analysis revealed a reasonable network structure between immune cell populations and differentially abundant taxa, previous attempts at standard correlations simply returned dense, isolated networks of immune cells and microbes with no significant connections between them (data not shown), likely due to masking of these associations by compositional interdependency within each dataset. Taken together, these results highlight the importance of considering compositional data structure to refine and contextualize conclusions but also to protect against type I and II error that may impinge upon downstream exploration.

This experiment simultaneously measured the population dynamics of the microbiome and multi-organ T-cell repertoire across the postnatal period, revealing novel observations about the co-evolution of the gut ecosystem and host adaptive immunity. Overall, the results of this study provide a rich characterization of developmental microbe-immune interactions throughout the early postnatal period. This included not only a description of the key microbial and immune cell populations that discriminate between different developmental milestones but also the relationships between functionally related bacterial consortia and their host counterparts in mucosal and central immune compartments. Integrating results from this experiment further supports the notion of a critical window for formative tuning of the immune system by key commensal taxa, whereby the presence of a normal, healthy microbiota from birth catalyzes and accelerates the immune maturation and stabilization of the organism.

Despite the many insights gleaned from this experiment, it had some limitations worth discussing. An important caveat of the present data is that it measures immune cells in the small intestine, which differ significantly from the colonic mucosa and are not directly in contact with the majority of commensal bacteria populating the gut. Indeed, the regional specialization of the intestinal immune system has been thoroughly reviewed (58, 112), and as noted above, there are some hypotheses that could not be tested given the spatial separation of immune cell and microbiome samples. However, the small intestinal immune system is known to harbor the vast majority of IELs compared to the colon, and the importance of this population is of high clinical interest in microbiome-related conditions and neurodevelopmental processes. Furthermore, while the small intestine harbors a much smaller microbial population than the colon (~10^3–^10^7^ versus 10^12^ microbial cells/gram, respectively (113)), their constituent communities are strongly correlated within individuals (114) and the small intestine microbiota supports many important functional niches involved in macronutrient digestion and absorption (115). Thus, developmental relationships between the colonic microbiota and the small intestine mucosal immune system still provide mechanistically valuable results. Further research should expand upon these findings to include measures of both the small intestine microbiota and the colonic LP to gain a complete understanding of microbe-immune interactions across development.

## Data Availability

The datasets presented in this study can be found in online repositories. The names of the repository/repositories and accession number(s) can be found below: https://www.ncbi.nlm.nih.gov/, PRJNA1170324.
