# Supplementary material for: Compositional maturation of the microbiome and adaptive immunity in the postnatal period
Source: Front Immunol. 2026 May 5;17:1772425. doi: 10.3389/fimmu.2026.1772425 (PMC13183565; doi:10.3389/fimmu.2026.1772425)
Supplement: Supplementary file 1 [file DataSheet1.pdf]

Supplementary Material

1.0 Supplementary Tables

***Table S1**: Antibody staining solution for lymphocyte surface markers and intracellular markers*

Marker	Fluoro chrome	Channel	Laser	Filter	Final [ ]	Dil.	Amt (μL)*	Panel I	Panel II
**CD45**	APC-Cy7	APC-Cy7	red	780/60	1ug/ml	1:400	0.5	X	X
**TCRB**	AF 700	AF 700	red	730/45	2.5ug/ml	1:200	1	X	X
**CD3e**	PE-Cy7	PE-Cy7	blue	780/60	1ug/ml	1:200	1		X
**CD4**	BB700	PerCP-Cy5.5	blue	695/40	1ug/ml	1:200	1	X	
**CD19**	BV605	BV605	violet	610/20	1ug/ml	1:200	0.5		X
**CD8a**	BV605	BV605	violet	610/20	1ug/ml	1:200	0.5	X	
**CD25**	eFluor 450	BV421	violet	450/40	1ug/ml	1:200	0.5	X	X
**L/D Aqua**	NA	BV510	violet	525/50	500X	1:500	1	X	X
**FoxP3**	APC	APC	red	670/30	1ug/ml	1:200	1	X	X
**RORγT**	AF 488	AF 488	blue	530/30	1ug/ml	1:200	0.4	X	X

** Amount in 50uL final volume*

Table S2: *Immune cell populations of interest for integrated analysis*

Population	Markers	Tissue	Stain
**Total T-cells (SS1)**	CD45+CD4(+/-)CD8(+/-)	SPLN, LPL, IEL	SS1
Helper T-cells	CD45+CD4+CD8-	All	SS1
T-regulatory cells	CD45+CD4+CD8-CD25(+/-)Foxp3+	All	SS1
Th17 cells	CD45+CD4+CD8-CD25(+/-)RORγT+	SPLN, LPL, IEL	SS1
Activated Th Cells	CD45+CD4+CD8-CD25+	All	SS1
pTregs	CD45+CD4+CD8- Foxp3+ RORγT+	IEL, LPL	SS1
Cytotoxic T-cells	CD45+CD4-CD8+	All	SS1
CD8+RORgT+ cells	CD45+CD4-CD8+CD25(+/-)RORγT+	THY	SS1
DP T-cells	CD45+CD4+CD8+	THY	SS1
DP RORγT+ cells	CD45+CD4+CD8+CD25(+/-)RORγT+	THY	SS1
DN T-cells	CD45+CD4-CD8-	THY	SS1
DN2/3 cells	CD45+CD4-CD8-CD25+	THY	SS1
DN3/4 cells	CD45+CD4-CD8-CD25-RORγT+	THY	SS1
**Total T-cells (SS2)**	CD45+CD3+CD19-	SPLN, LPL, IEL	SS2
αβ T-cells	CD45+CD19-CD3+TCRβ+	SPLN, LPL, IEL	SS2
**B-cells**	CD45+CD3-CD19+	SPLN, LPL, IEL	SS2

Table S3: List of Differentially Abundant (DA) taxa included in microbiome-immune cell network analysis								
**ASV**	**Kingdom**	**Phylum**	**Class**	**Order**	**Family**	**Genus**	**Species**	**full_name**
2650157	Bacteria	Bacteroidetes	Bacteroidia	Bacteroidales	Bacteroidaceae	Bacteroides	sp. HF-5287	Bacteroides_spHF-5287
2650158	Bacteria	Bacteroidetes	Bacteroidia	Bacteroidales	Bacteroidaceae	Bacteroides	sp. HF-5141	Bacteroides_spHF-5141
2763022	Bacteria	Bacteroidetes	Bacteroidia	Bacteroidales	Bacteroidaceae	Bacteroides	sp. M10	Bacteroides_spM10
2785531	Bacteria	Bacteroidetes	Bacteroidia	Bacteroidales	Bacteroidaceae	Bacteroides	sp. HF-162	Bacteroides_spHF-162
28111	Bacteria	Bacteroidetes	Bacteroidia	Bacteroidales	Bacteroidaceae	Bacteroides	eggerthii	Bacteroides_eggerthii
2755405	Bacteria	Bacteroidetes	Bacteroidia	Bacteroidales	Bacteroidaceae	Bacteroides	sp. CACC 737	Bacteroides_spCACC_737
28119	Bacteria	Bacteroidetes	Bacteroidia	Bacteroidales	Bacteroidaceae	Bacteroides	zoogleoformans	Bacteroides_zoogleoformans
290053	Bacteria	Bacteroidetes	Bacteroidia	Bacteroidales	Bacteroidaceae	Bacteroides	helcogenes	Bacteroides_helcogenes
47678	Bacteria	Bacteroidetes	Bacteroidia	Bacteroidales	Bacteroidaceae	Bacteroides	caccae	Bacteroides_caccae
329854	Bacteria	Bacteroidetes	Bacteroidia	Bacteroidales	Bacteroidaceae	Bacteroides	intestinalis	Bacteroides_intestinalis
246787	Bacteria	Bacteroidetes	Bacteroidia	Bacteroidales	Bacteroidaceae	Bacteroides	cellulosilyticus	Bacteroides_cellulosilyticus
2528203	Bacteria	Bacteroidetes	Bacteroidia	Bacteroidales	Bacteroidaceae	Bacteroides	sp. A1C1	Bacteroides_spA1C1
818	Bacteria	Bacteroidetes	Bacteroidia	Bacteroidales	Bacteroidaceae	Bacteroides	thetaiotaomicron	Bacteroides_thetaiotaomicron
820	Bacteria	Bacteroidetes	Bacteroidia	Bacteroidales	Bacteroidaceae	Bacteroides	uniformis	Bacteroides_uniformis
28113	Bacteria	Bacteroidetes	Bacteroidia	Bacteroidales	Bacteroidaceae	Bacteroides	heparinolyticus	Bacteroides_heparinolyticus
816	Bacteria	Bacteroidetes	Bacteroidia	Bacteroidales	Bacteroidaceae	Bacteroides		Bacteroides_NA_816
216816	Bacteria	Actinobacteria	Actinomycetia	Bifidobacteriales	Bifidobacteriaceae	Bifidobacterium		Bifidobacterium_NA_216816
1351	Bacteria	Firmicutes	Bacilli	Lactobacillales	Enterococcaceae	Enterococcus	faecalis	Enterococcus_faecalis
1350	Bacteria	Firmicutes	Bacilli	Lactobacillales	Enterococcaceae	Enterococcus		Enterococcus_NA_1350
1352	Bacteria	Firmicutes	Bacilli	Lactobacillales	Enterococcaceae	Enterococcus		Enterococcus_NA_1352
1578	Bacteria	Firmicutes	Bacilli	Lactobacillales	Lactobacillaceae	Lactobacillus		Lactobacillus_NA_1578
1598	Bacteria	Firmicutes	Bacilli	Lactobacillales	Lactobacillaceae	Limosilactobacillus	reuteri	Limosilactobacillus_reuteri
1622	Bacteria	Firmicutes	Bacilli	Lactobacillales	Lactobacillaceae	Ligilactobacillus	murinus	Ligilactobacillus_murinus
1584	Bacteria	Firmicutes	Bacilli	Lactobacillales	Lactobacillaceae	Lactobacillus		Lactobacillus_NA_1584
33958	Bacteria	Firmicutes	Bacilli	Lactobacillales	Lactobacillaceae			NA_NA_33958
33959	Bacteria	Firmicutes	Bacilli	Lactobacillales	Lactobacillaceae	Lactobacillus	johnsonii	Lactobacillus_johnsonii
1605	Bacteria	Firmicutes	Bacilli	Lactobacillales	Lactobacillaceae	Ligilactobacillus	animalis	Ligilactobacillus_animalis
584	Bacteria	Proteobacteria	Gammaproteobacteria	Enterobacterales	Morganellaceae	Proteus	mirabilis	Proteus_mirabilis
544645	Bacteria	Bacteroidetes	Bacteroidia	Bacteroidales	Odoribacteraceae	Butyricimonas	virosa	Butyricimonas_virosa
214856	Bacteria	Bacteroidetes	Bacteroidia	Bacteroidales	Rikenellaceae	Alistipes	finegoldii	Alistipes_finegoldii
1279	Bacteria	Firmicutes	Bacilli	Bacillales	Staphylococcaceae	Staphylococcus		Staphylococcus_NA_1279
1301	Bacteria	Firmicutes	Bacilli	Lactobacillales	Streptococcaceae	Streptococcus		Streptococcus_NA_1301
1326	Bacteria	Firmicutes	Bacilli	Lactobacillales	Streptococcaceae	Streptococcus	acidominimus	Streptococcus_acidominimus
1328	Bacteria	Firmicutes	Bacilli	Lactobacillales	Streptococcaceae	Streptococcus		Streptococcus_NA_1328
1814128	Bacteria	Firmicutes	Bacilli	Lactobacillales	Streptococcaceae	Streptococcus	halotolerans	Streptococcus_halotolerans
82348	Bacteria	Firmicutes	Bacilli	Lactobacillales	Streptococcaceae	Streptococcus	pluranimalium	Streptococcus_pluranimalium
328812	Bacteria	Bacteroidetes	Bacteroidia	Bacteroidales	Tannerellaceae	Parabacteroides	goldsteinii	Parabacteroides_goldsteinii

2.0 Supplementary Figures

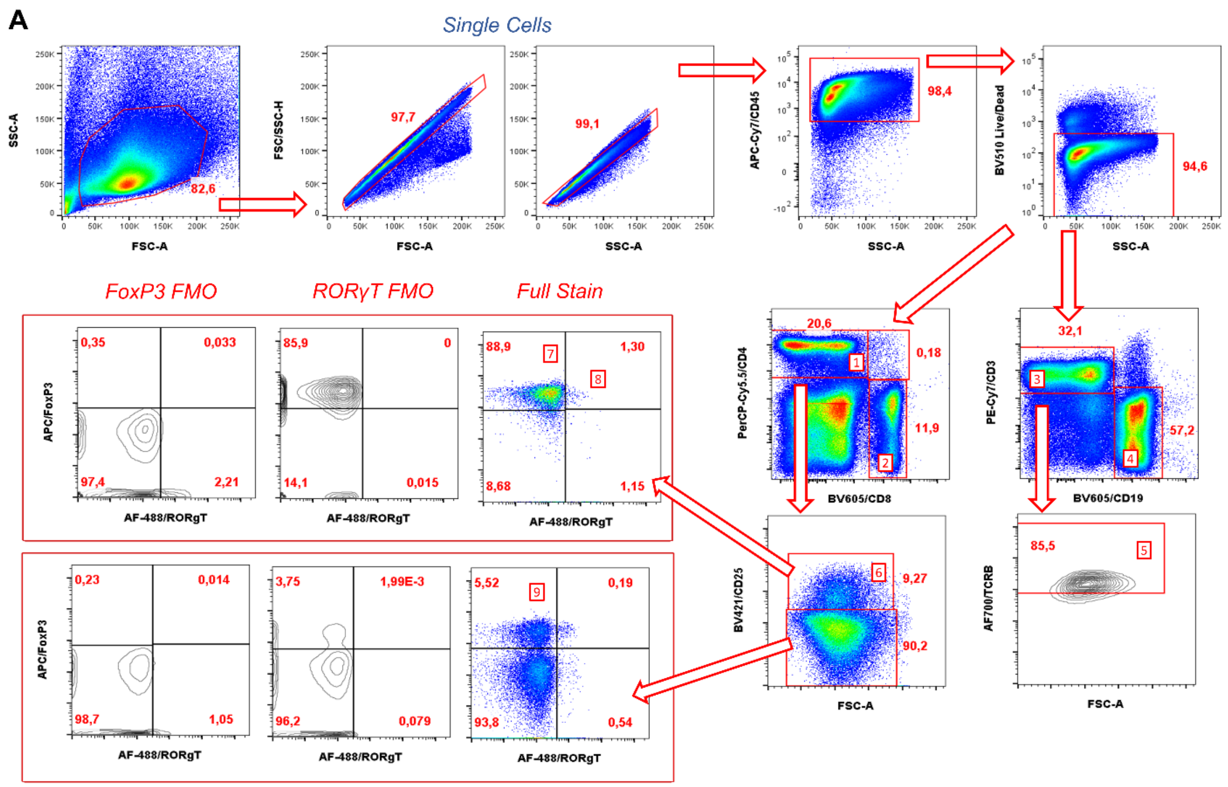

Supplemental Figure S1: Full gating strategy for splenocytes. Numbered gates represent variables of interest used for integrated analysis. Contour plots are shown to illustrate gate positioning based on FMO fluorescence values.

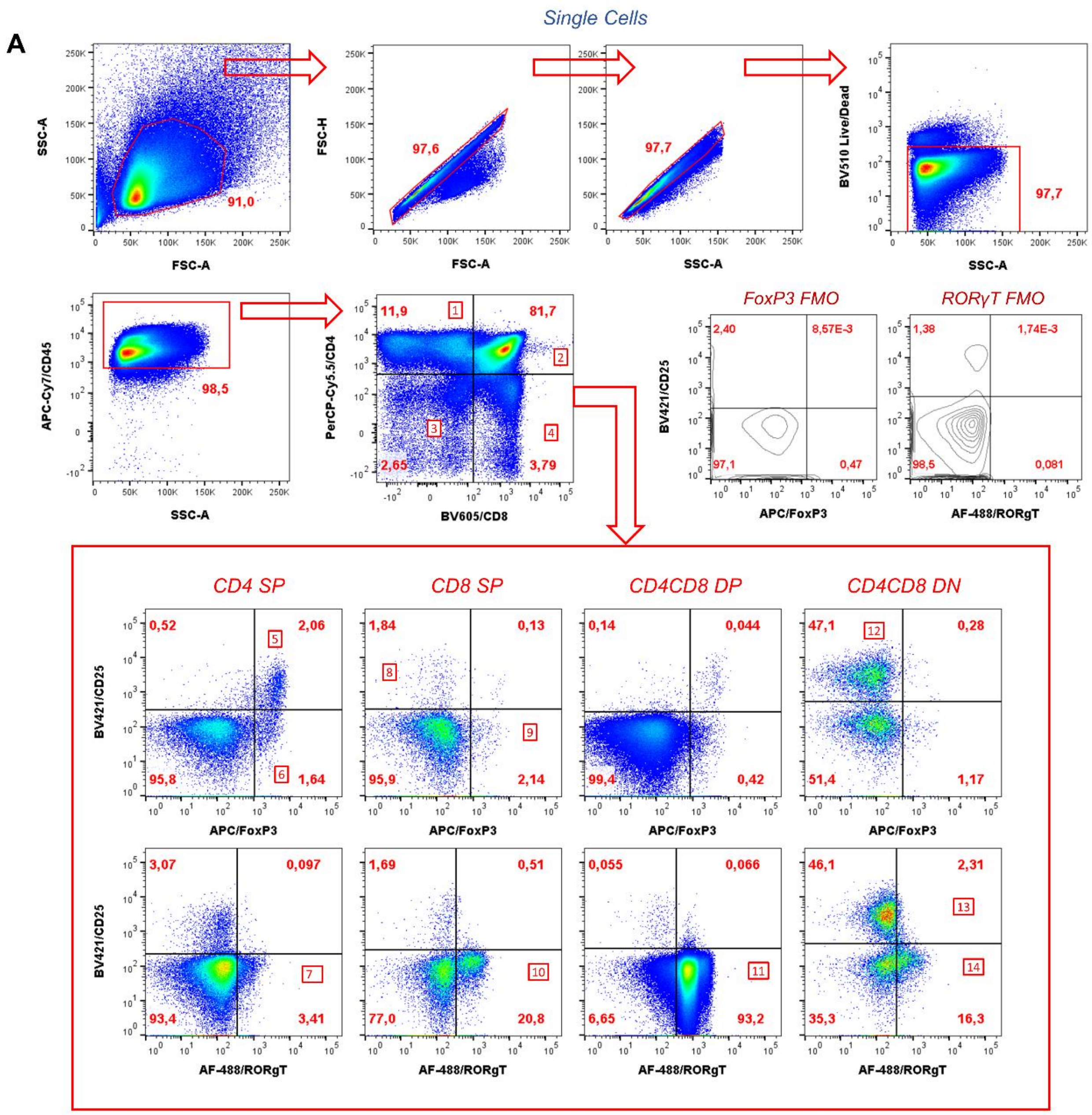

Supplemental Figure S2: Full gating strategy for thymocytes. Numbered gates represent variables of interest used for integrated analysis. Contour plots are shown to illustrate gate positioning based on FMO fluorescence values.

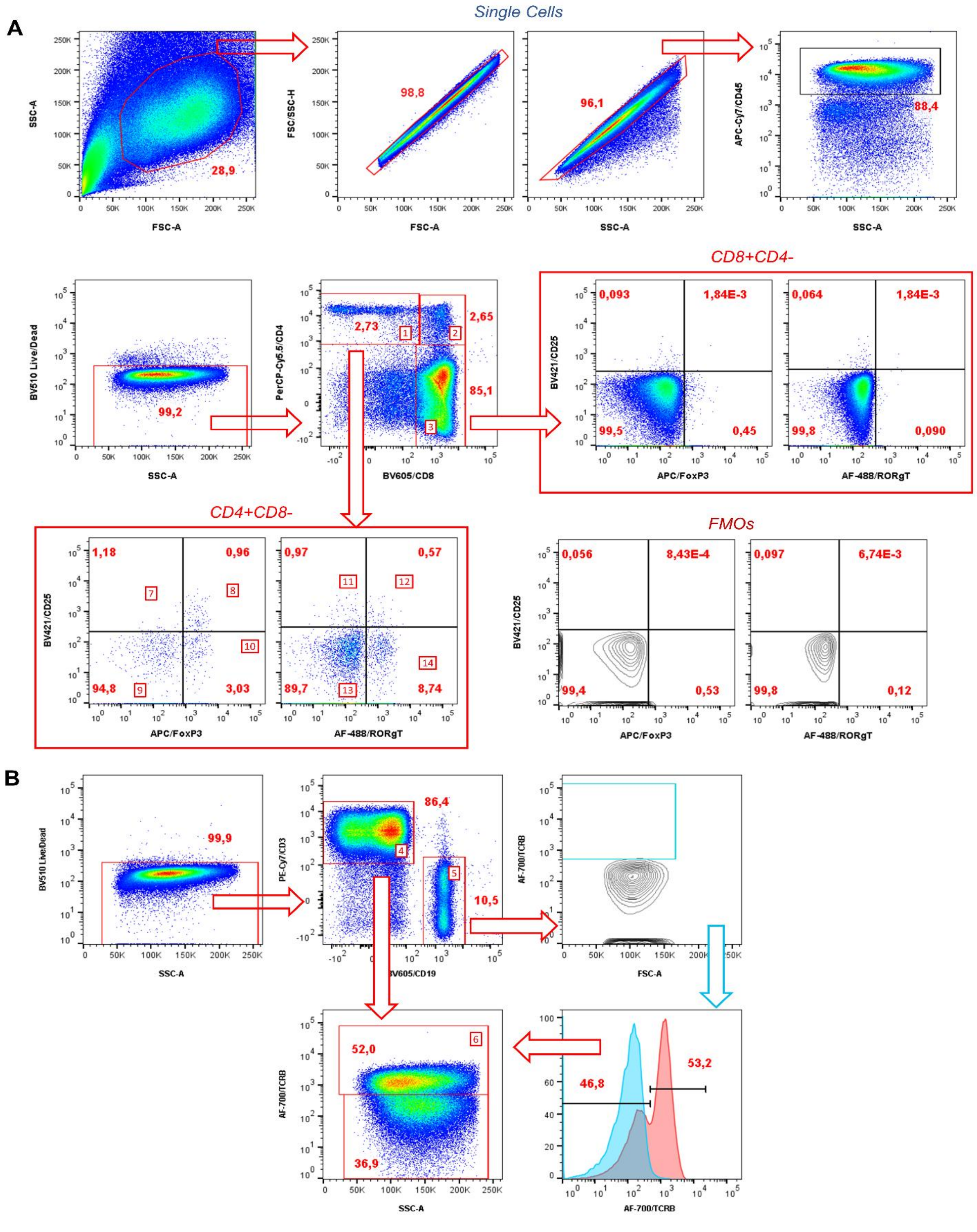

Supplemental Figure S3: Full gating strategy for IELs. A) shows the specific procedure for panel 1, B) shows the process for the second marker panel starting from a common gate on live cells using CD19+ B-cells as an internal "FMO" for TCRβ. Numbered gates represent variables of interest used for integrated analysis.

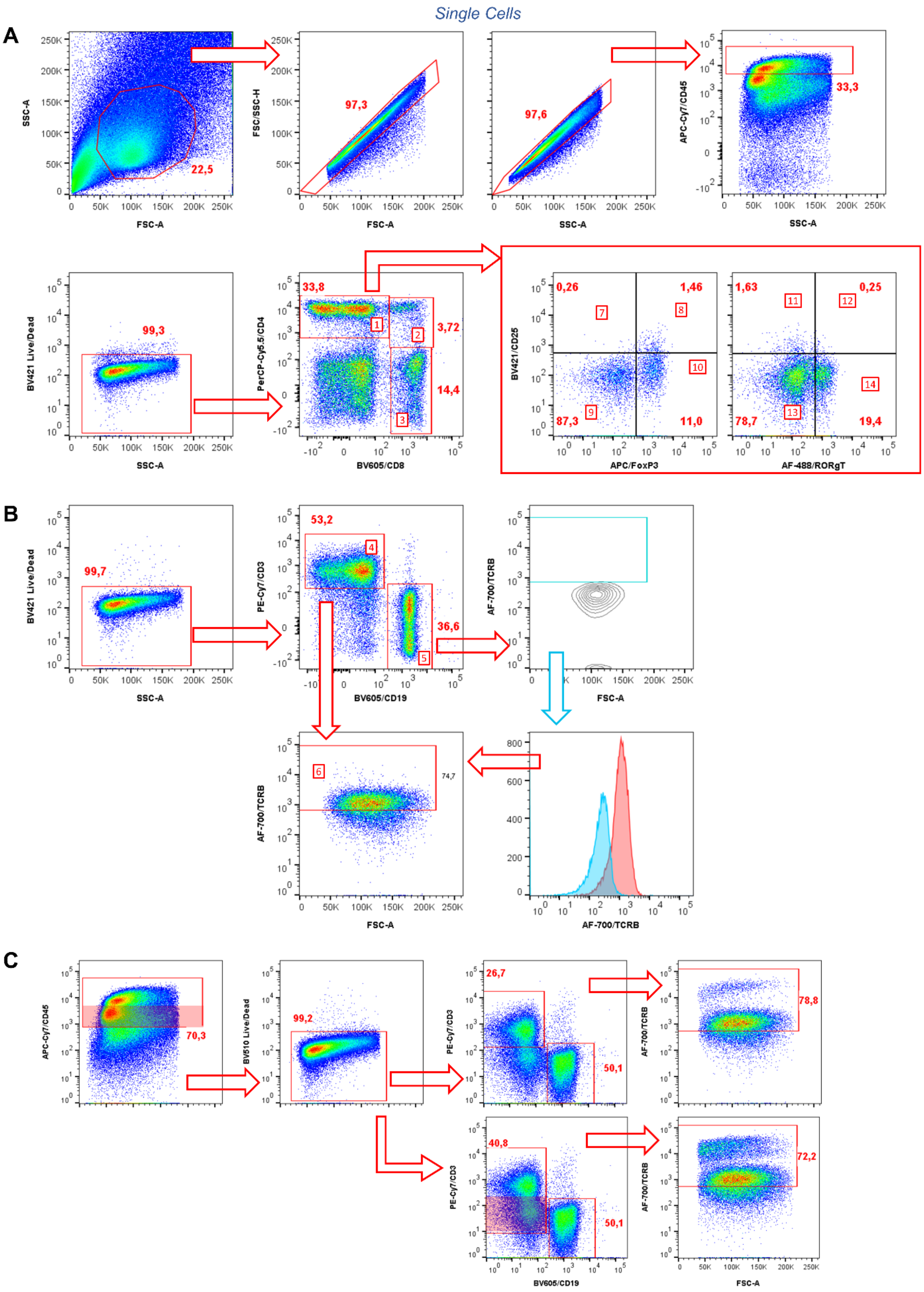
A
B
C
Single Cells
SSC-A
FSC-A
FSC/SSC-H
APC-Cy7/CD45
BV421 Live/Dead
PerCP-Cy5.5/CD4
BV605/CD8
BV421/CD25
APC/FoxP3
AF-488/RORgT
PE-Cy7/CD3
BV605/CD19
AF-700/TCRB
BV510 Live/Dead
22,5
97,3
97,6
33,3
99,3
33,8
3,72
14,4
0,26
1,46
87,3
11,0
1,63
0,25
78,7
19,4
99,7
53,2
36,6
74,7
70,3
99,2
26,7
50,1
78,8
40,8
50,1
72,2
1
2
3
4
5
6
7
8
9
10
11
12
13
14

Supplemental Figure S4: Full gating strategy for LPLs. A) shows the specific procedure for the first panel, while B shows the procedure for the second surface marker panel starting from a common gate on live cells using CD19+ B-cells as an internal "FMO" for TCRβ. Numbered gates represent variables of interest used in gated analysis. Panel C illustrates exclusion procedure to remove unidentified TCRβhi population from analysis.

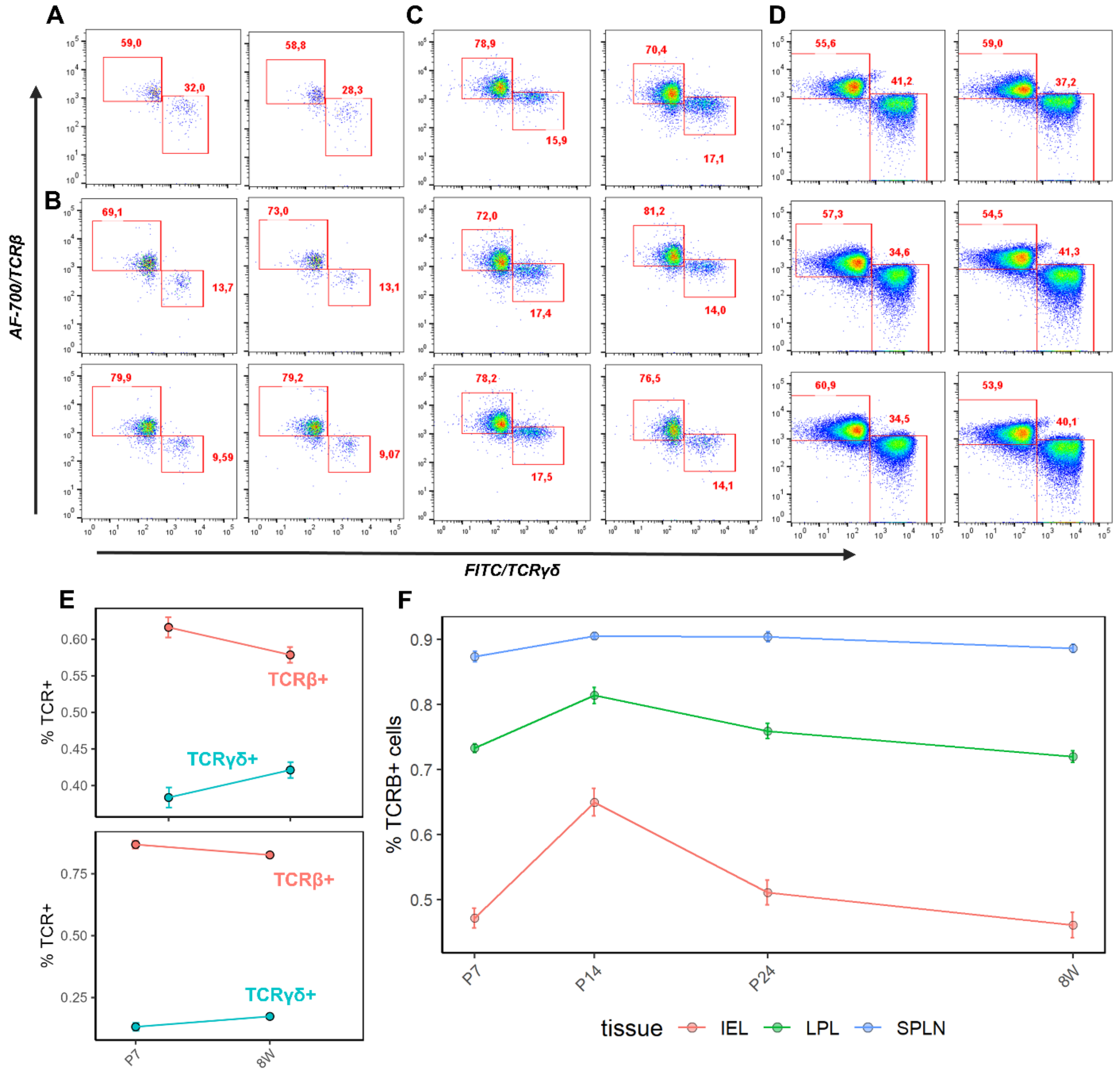

Supplemental Figure S5: Follow-up experiment measuring TCRβ+ and TCRγδ+ T-cells in IEL and LPL samples. Panel A) and B) show exemplary P7 samples stained with antibodies for each TCR subtype in the IEL (top) and the LPL (bottom). C) and D) show a second set of exemplary samples for adult mice in IEL and LPL, respectively. Panel E) shows mean dot plots of follow-up panel results in IEL (top) and LPL (bottom). F) shows original results from the second surface panel for comparison, which only distinguished TCRβ+/- cells within the CD3+ population.

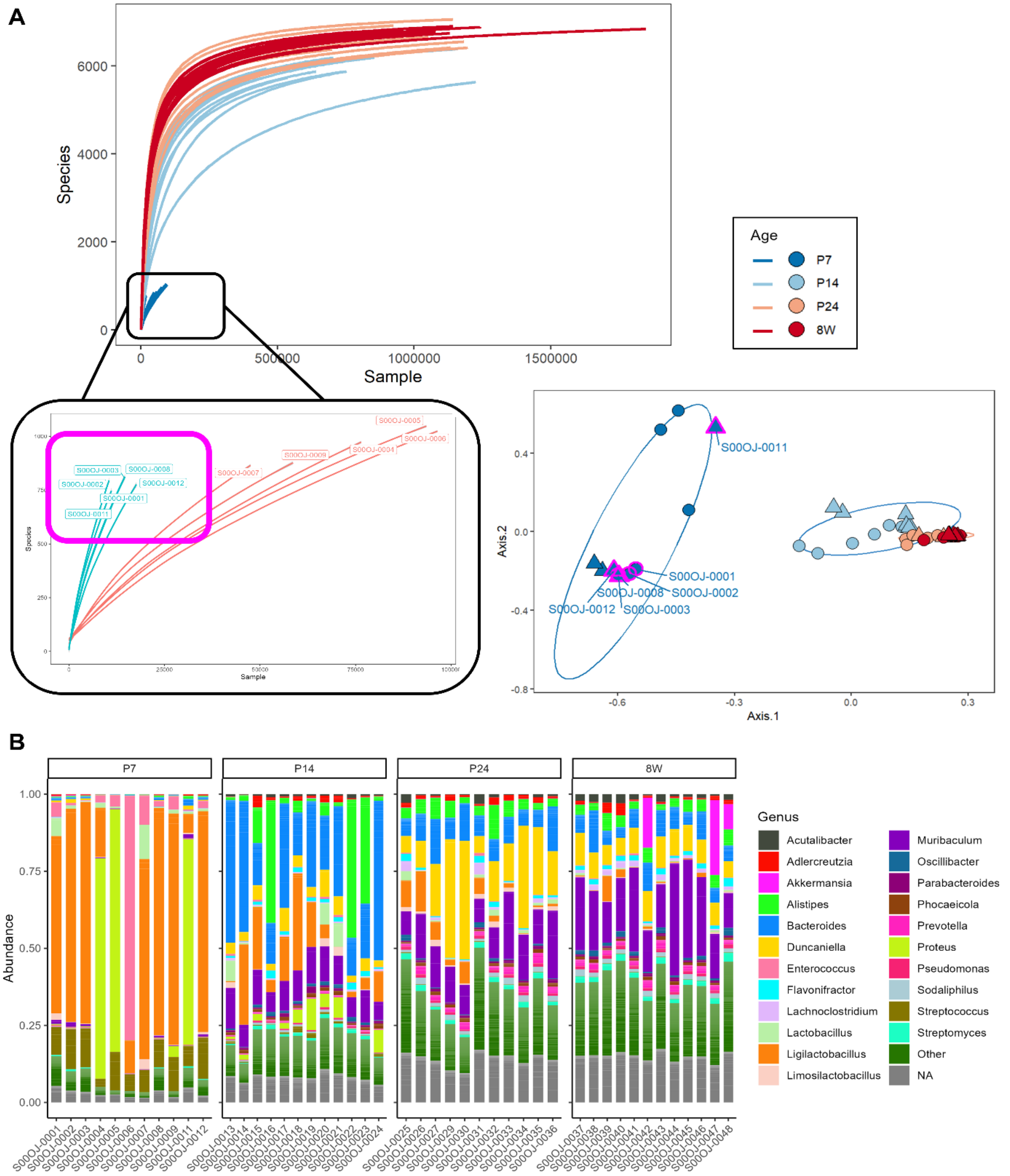

Supplemental Figure S6: additional visualizations for microbiome data QC and results. A) rarefaction curves across samples; while not all P7 samples appear to converge on a high-confidence richness estimate, but this does not appear to systematically impact microbiome composition. B) individual compositional bar plots for samples at the genus level.

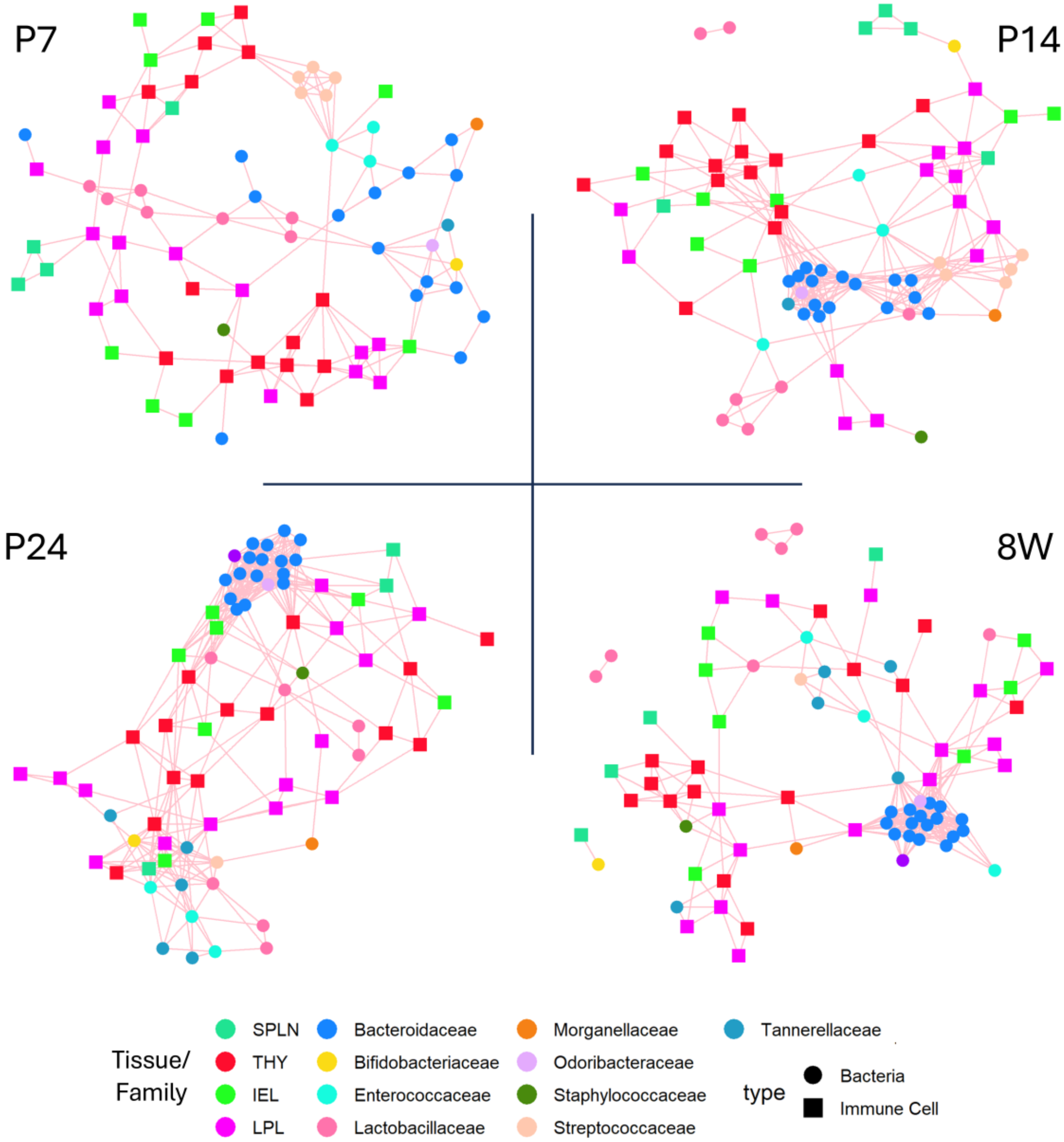

Supplemental Figure S7: Timepoint-specific propr networks of microbial taxa and multi-tissue immune cell subsets at P7, P14, P24 and 8W. Nodes are colored by tissue for immune cells (squares) and by Family classification for bacteria (circles). Displayed connections meet the FDR<0.05 equivalent criteria ($\rho > 0.45$) with isolated nodes removed.

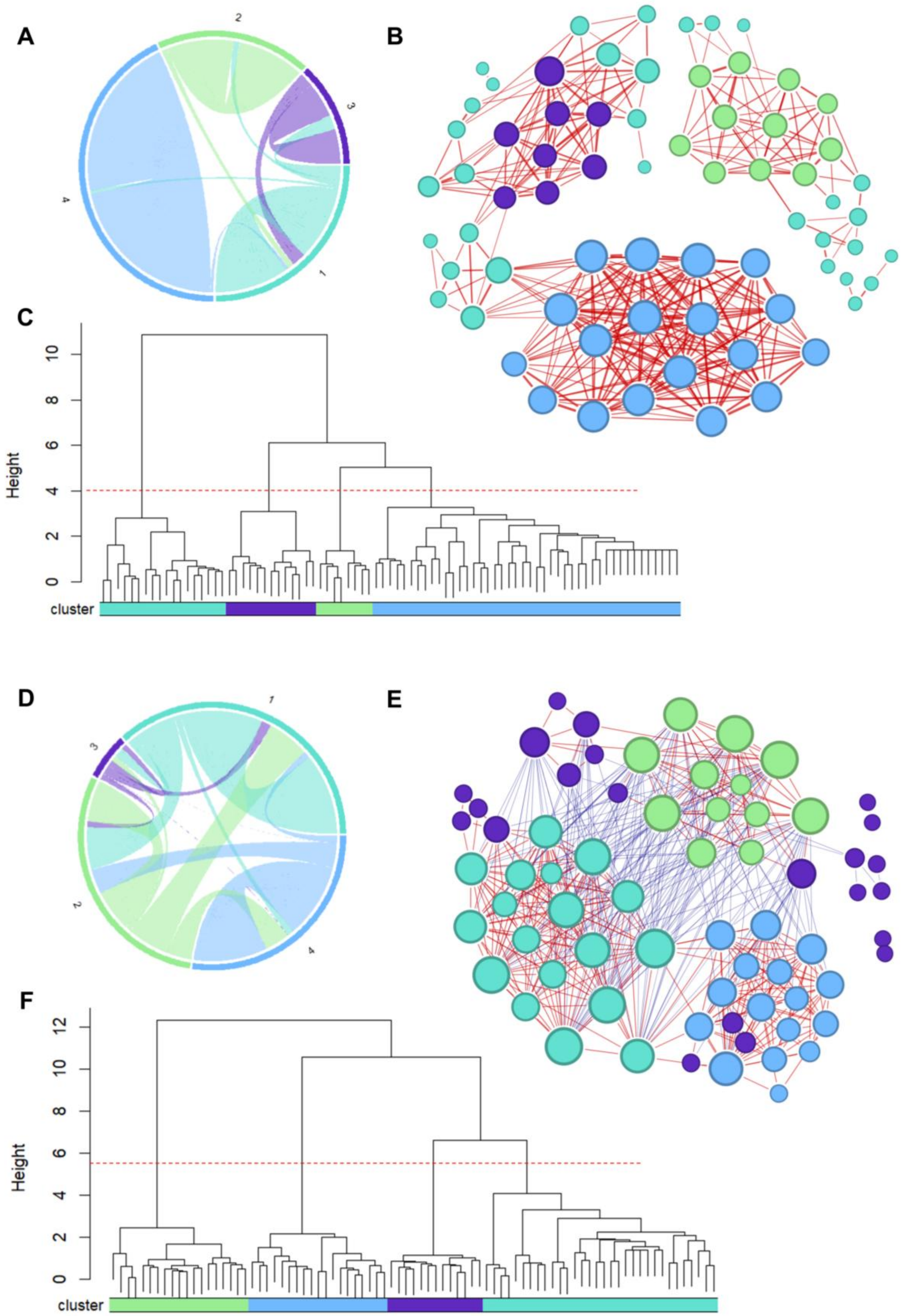
A
B
C
D
E
F
1
2
3
4
Height
cluster
0
2
4
6
8
10
12

Supplemental Figure S8: Hierarchical clustering of integrated immune cell and microbiome data. A) and D) show Circos plots representing links between clusters in propr and spearman rank correlation networks, respectively, represented graphically in B) and E). C) and F) show the clustering dendrogram with tips coloured according to cluster membership. Dashed line represents cut point in tree for cluster assignment.
